# Time Scales in Epigenetic Dynamics and Phenotypic Heterogeneity of Embryonic Stem Cells

**DOI:** 10.1371/journal.pcbi.1003380

**Published:** 2013-12-12

**Authors:** Masaki Sasai, Yudai Kawabata, Koh Makishi, Kazuhito Itoh, Tomoki P. Terada

**Affiliations:** 1 Department of Computational Science and Engineering, Nagoya University, Nagoya, Japan; 2 Department of Applied Physics, Nagoya University, Nagoya, Japan; 3 School of Computational Sciences, Korea Institute for Advanced Study, Seoul, Korea; 4 Okazaki Institute for Integrative Bioscience, Okazaki, Japan; CNAG - Centre Nacional d'Anàlisi Genòmica and CRG - Centre de Regulació Genòmica, Spain

## Abstract

A remarkable feature of the self-renewing population of embryonic stem cells (ESCs) is their phenotypic heterogeneity: Nanog and other marker proteins of ESCs show large cell-to-cell variation in their expression level, which should significantly influence the differentiation process of individual cells. The molecular mechanism and biological implication of this heterogeneity, however, still remain elusive. We address this problem by constructing a model of the core gene-network of mouse ESCs. The model takes account of processes of binding/unbinding of transcription factors, formation/dissolution of transcription apparatus, and modification of histone code at each locus of genes in the network. These processes are hierarchically interrelated to each other forming the dynamical feedback loops. By simulating stochastic dynamics of this model, we show that the phenotypic heterogeneity of ESCs can be explained when the chromatin at the *Nanog* locus undergoes the large scale reorganization in formation/dissolution of transcription apparatus, which should have the timescale similar to the cell cycle period. With this slow transcriptional switching of *Nanog*, the simulated ESCs fluctuate among multiple transient states, which can trigger the differentiation into the lineage-specific cell states. From the simulated transitions among cell states, the epigenetic landscape underlying transitions is calculated. The slow *Nanog* switching gives rise to the wide basin of ESC states in the landscape. The bimodal Nanog distribution arising from the kinetic flow running through this ESC basin prevents transdifferentiation and promotes the definite decision of the cell fate. These results show that the distribution of timescales of the regulatory processes is decisively important to characterize the fluctuation of cells and their differentiation process. The analyses through the epigenetic landscape and the kinetic flow on the landscape should provide a guideline to engineer cell differentiation.

## Author Summary

Embryonic stem cells (ESCs) can proliferate indefinitely by keeping pluripotency, i.e., the ability to differentiate into any cell-lineage. ESCs, therefore, have been the focus of intense biological and medical interests. A remarkable feature of ESCs is their phenotypic heterogeneity: ESCs show large cell-to-cell fluctuation in the expression level of Nanog, which is a key factor to maintain pluripotency. Since Nanog regulates many genes in ESCs, this fluctuation should seriously affect individual cells when they start differentiation. In this paper we analyze this phenotypic fluctuation by simulating the stochastic dynamics of gene network in ESCs. The model takes account of the mutually interrelated processes of gene regulation such as binding/unbinding of transcription factors, formation/dissolution of transcription apparatus, and histone-code modification. We show the distribution of timescales of these processes is decisively important to characterize the dynamical behavior of the gene network, and that the slow formation/dissolution of transcription apparatus at the *Nanog* locus explains the observed large fluctuation of ESCs. The epigenetic landscapes are calculated based on the stochastic simulation, and the role of the phenotypic fluctuation in the differentiation process is analyzed through the landscape picture.

## Introduction

Embryonic stem cells (ESCs) are pluripotent having the ability to differentiate into a variety of lineages, while in suitable culture conditions they proliferate indefinitely by maintaining pluripotency. These self-renewing ESCs are distinguished by the marker proteins including Sox2, Oct4 and Nanog (SON) [1]–[4]. SON are transcription factors (TFs) which directly or indirectly promote the expression of themselves by constituting an overall positive feedback network [5]–[10], among which Nanog is an essential factor working as a gatekeeper for pluripotency [11], [12]. Here, a remarkable feature is the large cell-to-cell variation of the level of Nanog in the self-renewing isogenic population of ESCs [13]–[15]. Since a distinct downregulation of Nanog is associated with the differentiation of ESCs into mesendoderm or neural ectoderm lineages [16], the heterogeneous Nanog expression can be intimately related to the process of fate decision of individual cells [14], [17]. The molecular mechanism and biological implication of this phenotypic fluctuation of ESCs, however, have not yet been clarified. In this paper we address this problem by constructing a model of the regulatory network of core genes in mouse ESCs.

One can figure out, at a glance, several scenarios which may explain the phenotypic heterogeneity. A simple scenario relies on the possible enhancement of fluctuation of the signal received by a cell: Since the reception of factors such as leukemia inhibitory factor (Lif) by a cell is stochastic, it necessarily bears fluctuation, which might be enhanced through the signal cascade to stochastically activate *Nanog* [18]. The other possible mechanism is based on the presumed self-activation of *Nanog* [6], [19], which may lead to the fluctuating pulsative expression of Nanog [14], [20]. With these mechanisms, however, another key factor, Oct4, should also exhibit the large fluctuation since *Oct4* is activated by the reception of Lif and the Oct4 expression is maintained through mutually activating interactions among SON. Contrary to this expectation, the observed expression of Oct4 is rather homogeneous [14], [17]. A possible resolution of this inconsistency is to assume that some unknown factors which can bind to the *Oct4* locus suppress fluctuation of the Oct4 expression [20]. There has been, however, no direct experimental observation yet for the existence of such regulatory factors, and therefore, in this paper we look for the other mechanism without relying on this assumption.

For modeling the gene regulatory dynamics, not only the topological wiring diagram among genes but also the rates of reactions in the regulatory network should be quantified. These estimated rates, however, have very different values depending on the type of reactions, and hence it is strongly desired to develop the theoretical framework to treat effects of coexistence of the distributed timescales [21], [22]. In simple bacterial cells, for example, the DNA-protein binding/unbinding is often much faster than the protein-copy number change, so that the fast DNA-state change can be regarded as equilibrated and the dynamical interference between the fluctuation of gene switching and the fluctuation of protein-copy number can be neglected. By borrowing the wording from condensed-matter physics, this separation of fast and slow processes should be referred to as the “adiabatic” separation. Theoretical studies have shown that when the adiabatic limit is not the case, the kinetic flow of the coupled stochastic dynamics of gene switching and protein-copy number change is described as “eddy current” [23], which gives rise to a variety of unexpected dynamical effects in gene regulation [23]–[28]. Indeed, it has been suggested that the transition of *Bacillus subtilis* into the competence period should be due to the non-adiabatic gene switching in the excitatory self-activating gene network [29]. In eukaryotic cells, processes of gene switching are much more complex including the assembly of transcriptional apparatus (TA) [30]–[33], the transition from the poised state of TA to the elongation state [34], chemical modifications of nucleosomes [35]–[39], and the structural reorganization of chromosomes [40]–[42]. Such epigenetic change of the gene state can be much slower than the bacterial DNA state change, and their timescales are often comparable with or longer than the timescale of the protein-copy number change, so that the non-adiabatic effects should play significant roles in eukaryotic cells.

Many marker genes of ESCs have been identified [43], among which SON regulate many other genes, and hence, the SON network has been regarded as the central network to maintain pluripotency [8], [9], [43]. Models of the core SON network of ESCs have been developed [14], [16], [20], [43], [44], but all of these models have been based on the assumption that the gene state is determined by the fast equilibrated binding/unbinding of TF to/from the gene locus: The assumption of the adiabatic limit has been adopted in all the previous models and the slow non-adiabatic switching dynamics has not been explicitly taken into account. In this paper, we discuss ESCs by focusing on the non-adiabatic effects, the effects of slow epigenetic processes, and we propose a hypothesis that the non-adiabatic switching in the core gene-network explains the large fluctuation of Nanog expression. By using the landscape picture, we discuss the roles of this non-adiabatic switching in the cell-fate decision of ESCs.

## Results

Before starting the explanation of the simulated results, we briefly explain the interaction network among genes considered in the present model and discuss the dynamics of each gene in the network in subsection *Gene network and epigenetic dynamics*. Coexistence of multiple timescales in the eukaryotic gene dynamics is the focus of the present study.

### Gene network and epigenetic dynamics

#### The interaction network

**Figure 1:**
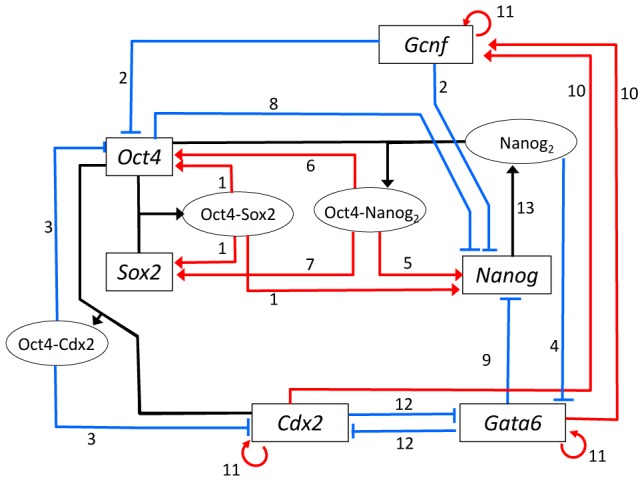
The network model. Interactions among marker genes of ESCs, *Sox2*, *Oct4*, and *Nanog*, and lineage-specific genes, *Gata6*, *Cdx2*, and *Gcnf* are shown with black lines for formation of complexes of factors synthesized from the corresponding genes, red lines for activating the target genes, and blue lines for repressing the target genes. Experimental data used to infer the interactions are designated by numbers on lines, which are explained with the corresponding numbering in subsection *Inference of the network from experimental observations* in section *Methods*.

Interactions among genes have been inferred from observations on how the expressions of genes are correlated with each other and how one factor binds to the genetic locus of the other factor. It is not straightforward, however, to identify each elementary interaction embedded in the complex web of regulation. Indeed, consensus has not been obtained on the role of *Nanog*: Though both *Sox2* and *Oct4* loci have the Nanog binding sites [6] and the assumption of positive regulation of *Sox2* and *Oct4* by Nanog is reasonable [5], [8], the weak correlation of levels of Sox2 and Oct4 to the heterogeneous Nanog expression has cast doubts on the direct positive regulation by Nanog [11], [45]. The network model of Fig. 1 is based on the assumptions that Nanog directly activates *Sox2* and *Oct4*. We show that even with such assumptions, the weak correlation of Sox2 and Oct4 to the heterogeneous Nanog expression can be explained when epigenetic dynamics is considered explicitly.

In the model of Fig. 1, the representative lineage-trigger genes are also taken into account. Mouse ESCs can directly differentiate into either of trophectoderm, primitive endoderm, or primitive ectoderm [10]. Though majority of ESCs differentiate into primitive ectoderm under the condition of the minimal external stimuli [46], the core genes to guide ESCs to primitive ectoderm are still elusive, and hence in this paper, in order to analyze the effects of non-adiabaticity on differentiation, we focus on the rest two routes to trophectoderm and primitive endoderm. Lineage specific factors, Cdx2 for trophectoderm and Gata6 for primitive endoderm, repress expression of SON [1], [2], [47] by their direct binding to the enhancers [10], [47] or the indirect action through Gcnf [48], while SON repress *Cdx2* and *Gata6* [13], [47], [49]. The network of Fig. 1 resembles those proposed in [10] and analyzed in [50]. See subsection *Inference of the network from experimental observations* in section *Methods* for the explanation of the experimental data used to infer the network.

#### Epigenetic dynamics

**Figure 2:**
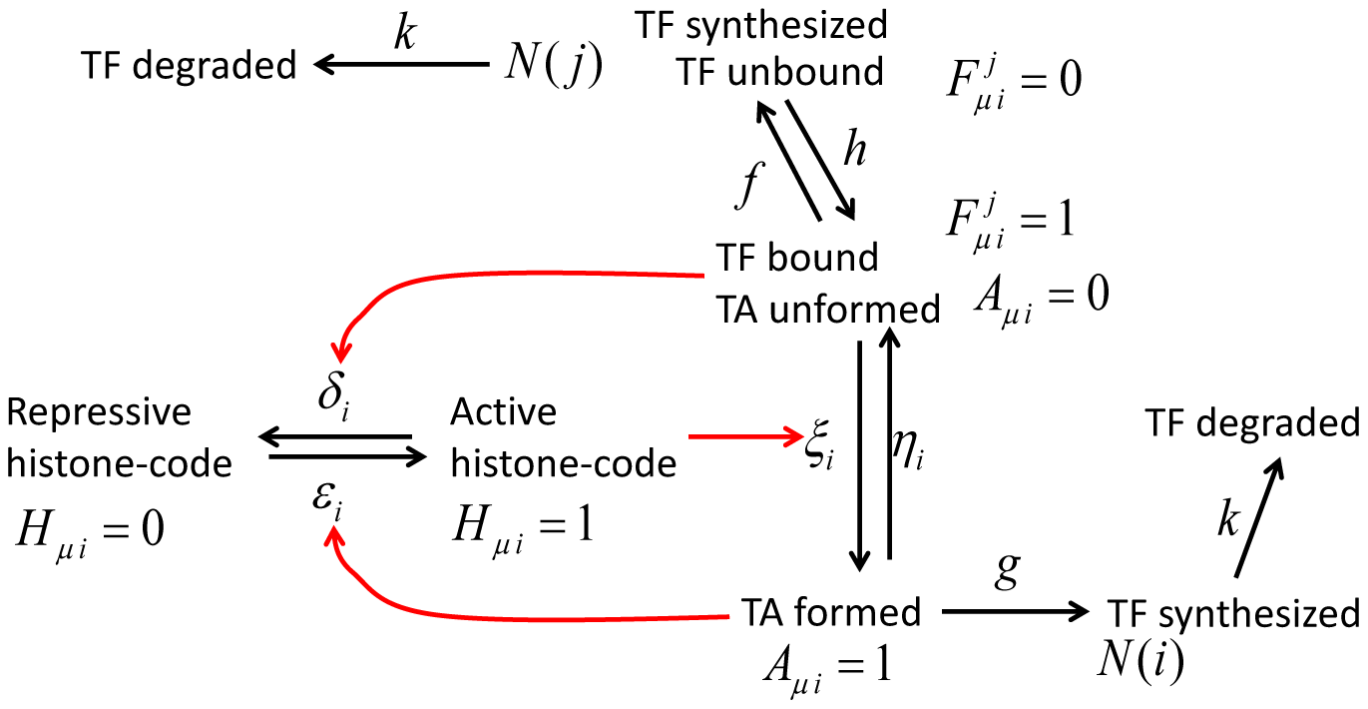
A sketch of the epigenetic switching in the model. The state of the allele 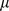 of gene 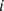 is described by 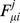, 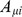, and 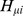. Binding of TF (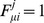) triggers the formation of TA (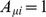), which enables synthesis of the 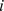
th TF to increase its copy number 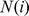. Rates of reactions, 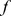, 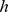, 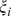, 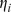, 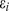, 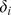, 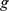, and 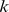, are written on the corresponding arrows. 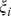 is large when the histone code is active for transcription (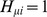), 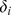 is large when the bound repressor at 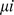 recruits the enzymes to rewrite the histone code, and 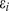 is large when the bound activator TF recruits the necessary enzymes (red arrows). The adiabaticity parameters are defined by 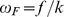, 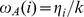, and 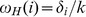. See subsection *Reactions in the model* in section *Methods* for the precise definition of reactions and rates and *Parameters* in *Methods* for the values of rate constants.

Shown in Fig. 2 is a sketch of how each gene is regulated through epigenetic dynamics in the model: Changes in the gene activity are triggered by binding/unbinding of TF to/from DNA. We write 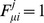 (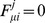) when the 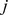 th TF is bound on (unbound from) the regulatory region of DNA at the locus of allele 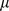 of gene 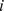, where each gene consists of two alleles 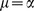 and 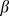. When the activator TF binds to the locus, the subsequent assembly of other molecules should take place to form a TA. The TA becomes ready for transcription when the chromatin structure is altered to accommodate the assembled factors and those factors are chemically suitably modified. For simplicity of description, we refer to the combined multiple processes to make TA active as “TA formation” and the processes to make TA inactive as “TA dissolution”. We write 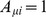 when the TA is formed and becomes ready for transcription, and 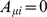 when the TA is dissolved and not ready for transcription. Changes in 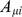 are regulated not only by the direct interactions among TFs and other factors on the gene locus but also by methylation or acetylation of nucleosomes at around 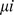. Since the ensemble of nucleosomes are methylated or acetylated cooperatively to form a bistable switch [35], [39], we write the state of the ensemble of nucleosomes, i.e., the histone code, as 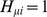 when the histone code promotes formation of the active TA, and 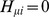 when it is inhibitory from transcription. It has been recently observed that only a single allele of *Nanog* is active in ESCs cultivated with Lif though two alleles can be active for other genes [51], which indicates that the Nanog expression is regulated through the large scale organization of chromosome architecture. This allelic regulation is represented in the model by fixing the values as 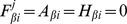 and only the dynamical change of the allele 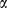 is considered for 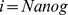.

The rate of binding of the 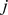 th TF (the rate to turn 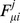 from 0 to 1), 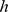, depends on the copy number of the 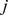
th TF, 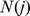. The rate to change 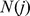 depends on the rate of synthesis of the TF, 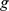, the average burst size, 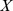 (see Eq.1 for the definition of burst synthesis of proteins), and the degradation rate of the TF, 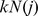, where the synthesis rate 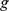 depends on whether the TA is ready for transcription (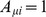) or not (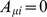). Rates of the TA formation/dissolution (rates to change 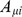) depend on the TF binding status 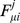 and the histone code 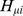, and rates to change 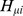 should depend on 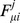 and 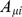 because the enzymes to rewrite the histone code are recruited by the TF bound on the locus. In this way, the model has the hierarchically interrelated processes to change variables, 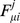, 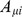, 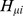, and 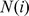.

These different processes should have different timescales; 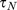 for the protein-copy-number change, 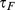 for binding/unbinding of TF, 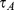 for formation/dissolution of a TA on chromatin, and 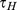 for modification of the histone code. Their precise values are not known, but the histone code is often heritable across generations of cell cycle [35] and modification of the histone code is most frequent at the late phase of mitosis [52], [53]. Therefore, when the DNA methylation does not inhibit the histone-code modification, 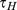 should be larger than or near to the period of cell cycle, 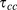, as 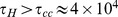 sec. When DNA is methylated, on the other hand, the timescale to make the histone code active (the timescale to turn 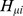 from 0 to 1) could be much longer as 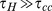, so that in general, 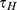 depends on the chemical state of DNA of each gene as 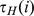. Since methylation of DNA is erased after fertilization, we can expect that DNA is not yet methylated at most of the gene loci in the early phase of differentiation we treat in the model. After the *de novo* methylation in the developmental process, the distribution pattern of methylated regions of DNA often lasts through the lifetime of the organism, showing its timescale is very long [54]. Therefore, we here do not consider the dynamics of DNA methylation explicitly, but treat it as a given static condition in our model.

From the observed rate of decrease of the amount of Oct4 [4] or Nanog [16], 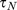 can be estimated as 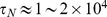 sec, which indicates 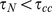. In many eukaryotic cells, the fast binding/unbinding of TF is necessary for cells to respond to fast environmental fluctuations, and hence we assume that also in ESCs its timescale 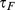 is much shorter than the timescale of protein-copy number change 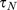 as 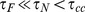. The timescale of TA formation/dissolution (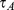) is not known but we assume that the timescale is correlated with complexity of the process. Building a TA with DNA looping and recruitment of molecules is more complex than the TF binding, so that we assume 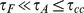. For the simple cases, we expect 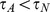, but when the large scale chromatin modification or the chromosome reorganization is necessary for building the TA, 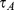 may become as large as 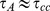. We write 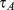 as 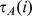 to emphasize its dependence on the type of gene.

The turnover time of TF binding/unbinding is 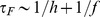, where 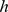 is the rate of binding and 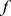 is the rate of unbinding. The turnover time of TA formation/dissolution is 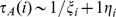, where 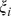 is the rate of formation and 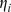 is the rate of dissolution. The turnover time of the histone-code modification is 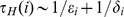, where 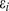 is the rate to turn the histone code positive for transcription and 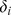 is the rate to turn the histone code repressive for transcription. We can expect in many cases that the balance is achieved between forward and backward processes for the dynamically fluctuating phase of the early differentiation, or in other words, 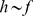, 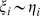, and 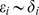. We therefore approximate times for turnover as 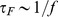, 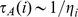, and 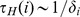. For 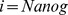, as will be discussed later in *Results* section, we have 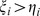 for the parameterization quantitatively consistent with the experimentally observed data, and hence we use the relation 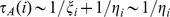 also for the *Nanog* locus.

Here, we define “adiabaticity” as the ratio of the rate of the process to the rate of protein copy-number change; adiabaticity is larger (smaller) than 1 when the process is adiabatic (non-adiabatic). We use three sets of adiabaticity parameters, 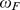, 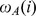, and 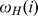. Using the rate constant of the protein degradation 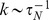 as a measure, the rate of unbinding of TF from DNA, 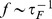, should be larger than 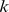, so that the adiabaticity of TF binding/unbinding is 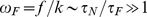. The rate to change the histone code from active to repressive state, 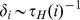, leads to the smaller adiabaticity as 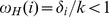. The rate of dissolution of TA should depend on the gene as 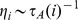. We expect that the corresponding adiabaticity parameter is 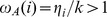 when the large scale reorganization of chromosome is not necessary, but 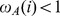 otherwise. Thus, 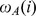 is a key parameter to determine the dynamical features of the whole switching process of the gene.

As in the previous works [29], [30], we do not explicitly consider mRNA and treat transcription and translation as one combined process. In the same spirit, we do not explicitly consider processes of the post-translational modification of TF, the transport of TF, or the actions of micro RNA. Though regulations through these processes can indeed affect the noise level [55]–[57], we here focus on the transcriptional regulations to clarify the effects of non-adiabatic gene switching. With this simplification, the states of six genes in Fig. 1 are described by 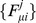, 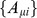, and 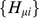, and the coupled stochastic dynamics of genes and 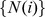 is simulated by using the Gillespie algorithm [58]. In this way, the present model has the resolution intermediate between the simplistic Boolean models [59] and the models which integrate the further detailed molecular processes. Through this simplified modeling and simulations, we propose a hypothesis that the hierarchically designed adiabaticity, 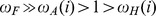 or 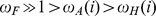, decisively affects the self-renewal of ESCs and differentiation. In *Parameters* subsection of *Methods* section of this paper, values of 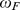 and 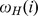 are estimated by referring the experimental data, but the value of 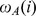 is largely undetermined. In the following part of *Results* section, we focus on the effects of varying 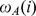 on cell dynamics.

### Phenotypic heterogeneity

We first discuss ESCs in media containing Lif and other agents. Lif activates *c-Myc* [60], which activates SON by keeping the histone code of lineage-specific genes repressive [34]. We simulate this culture by adopting the null rate for turning the histone-code active as 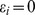 for 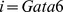, 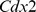 and 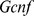 (See Fig. 2 and *Methods* for the definition of parameters).

**Figure 3:**
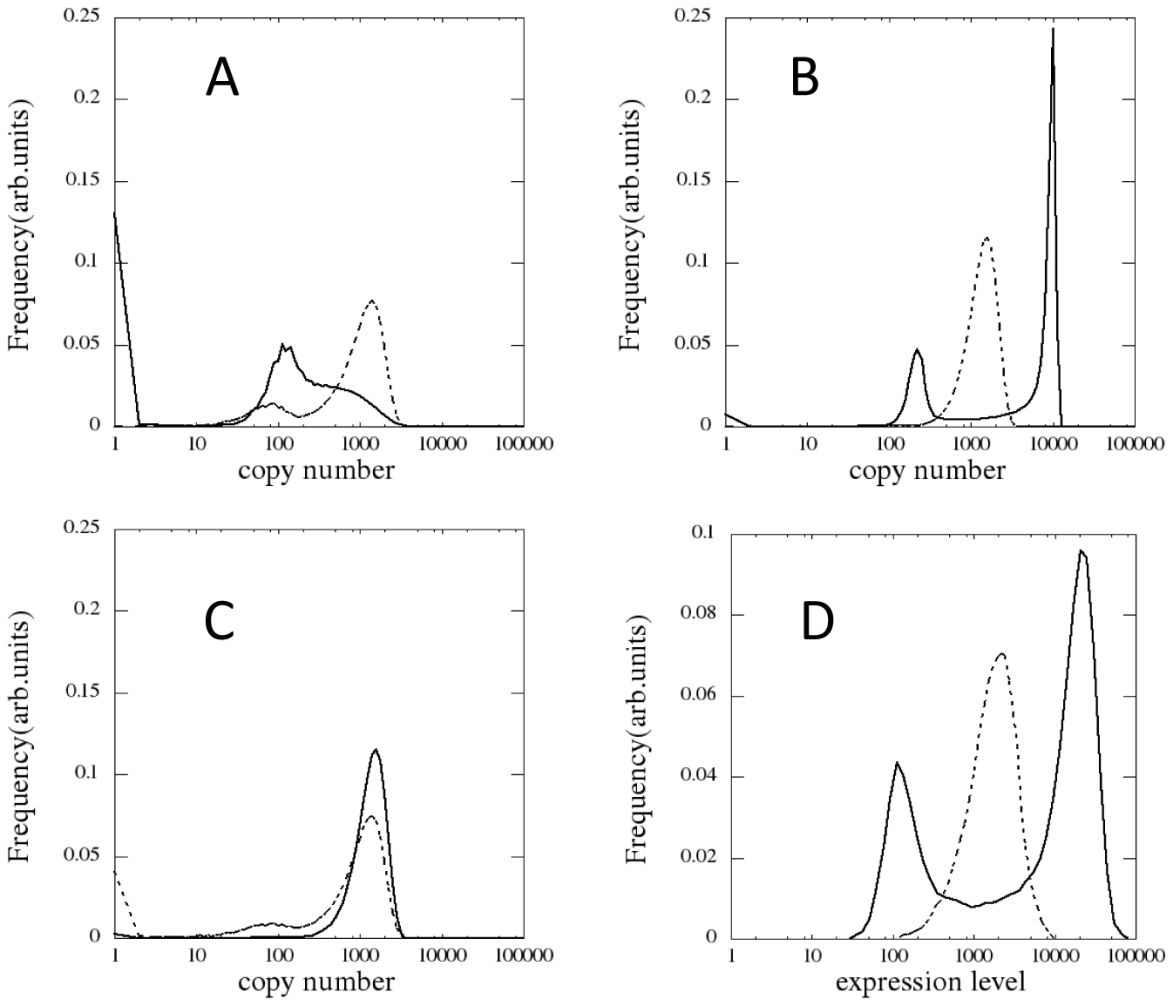
Distributions of the expression level of SON in the isogenic population of ESCs. Distributions of the simulated copy number of (*A*) Nanog (real line) and Oct4 (dashed line) with fast *Nanog* switching, (*B*) Nanog (real line) and Oct4 (dashed line) with the slow *Nanog* switching, and (*C*) Sox2 with the slow (real line) and fast (dashed line) *Nanog* switching. (*D*) The observed population of cells in a culture as a function of their expression level (in arbitrary units) of Nanog (real line, taken from Fig. S3 of [14]) and that of Oct4 (dashed line, modified from Fig. 6A of [14]). (*A*–*C*) Simulated by sampling 10,000 cells during 11.5 days with 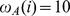 for all 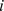 for the fast *Nanog* switching, and with 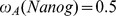 and 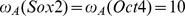 for the slow *Nanog* switching. For convenience of the graphical representation, the populations at the zero expression level (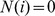) are binned with the data of 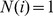.

First simulated is the case that the formation/dissolution of TA is adiabatic with 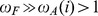 with 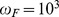. As a typical value to satisfy this inequality, we use 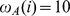 for all 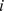. Distributions of the expression level of SON simulated with this parameterization are shown in Figs. 3A and 3C. We can see that the simulated distribution of the expression level of Nanog shows a single peak and the simulated distributions of Sox2 and Oct4 are double peaked at their finite values of expression level with some additional populations at the zero expression. These features are different from those observed in experiments: Compared are the distributions of cell population in a culture plotted as functions of the expression level of SON. The observed distribution of Oct4 is single peaked (Fig. 3D) [14], the distribution of Sox2 is similar to that of Oct4 [14], and the observed distribution of Nanog shows two peaks (Fig. 3D) [11], [14]. The observed two-peak distribution of Nanog indicates that the fluctuation of Nanog is dominated by transitions between two states; the high-Nanog (HN) and low-Nanog (LN) states [11], [33]. The simulated Nanog distribution with the adiabatic TA formation/dissolution apparently disagrees with this observed two-peaked Nanog distribution.

**Figure 4:**
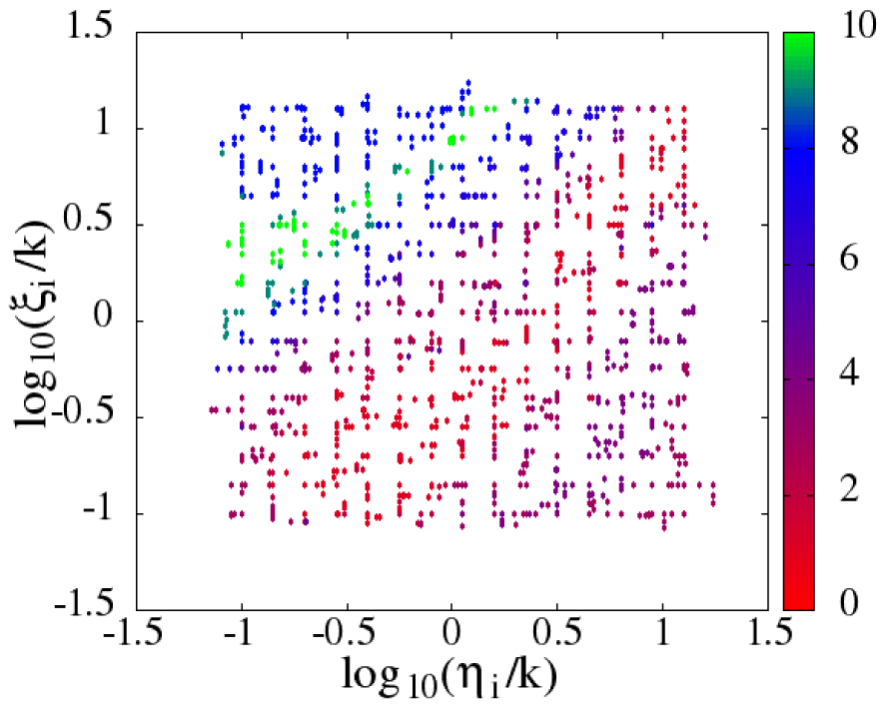
Search for a range of parameter set 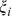 and 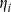 for 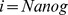. The ability of the simulated trajectories to reproduce the experimentally observed data of distributions of the expression level of SON is evaluated by the score 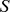 which is defined by Eq.20 in *Methods* section. The score was evaluated for each of 1,125 parameter sets scattered on the two-dimensional plane of 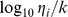 and 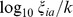 for 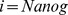, where 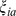 is a conditional value of 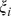 explained in *Methods* section and 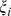 is the rate of TA formation. 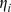 is the rate of TA dissolution, and 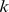 is the rate coefficient of protein degradation.

The assumption of the adiabatic TA formation/dissolution with 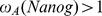 used in the above simulation is questionable when we consider the following features of Nanog expression: First, the TA of Nanog consists of the fairly large (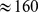
kb) region of DNA [61], which should make the formation/dissolution of TA a rather complex process. Second, the allelic regulation of Nanog [51] indicates that the chromosome organization on the nuclear scale regulates the Nanog expression. This observation is also consistent with the recent finding that the loci of genes of the pluripotent factors are spatially in proximity to the *Nanog* locus in an ESC-specific manner [62], indicating that the nuclear scale organization of chromosomes is involved in the activation of *Nanog* in ESCs. For such complex and spatially extended processes for TA at the *Nanog* locus, it should be reasonable to assume that the timescale of TA formation/dissolution is as long as the cell cycle period. To find the plausible values for the rate of TA formation (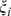) and the rate of TA dissolution (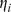) at the 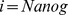 locus, we performed a massive parameter search by generating more than 1,000 scattered points on the two-dimensional plane of 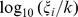 and 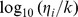 with 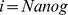. The score for each of generated parameter sets was calculated by averaging 10,000 trajectories simulated with the corresponding parameter set, where the score is the number of the experimentally observed features that the simulated data reproduced. The features used to count the score include (1) bimodality of the distribution of expression level of Nanog, (2) the ratio of the copy-number at the HN state to that at the LN state, (3) the ratio of the peak height at the HN state to that at the LN state, (4) the single-peak distribution of expression level of Oct4, and so on. The score calculated in this way is plotted in Fig. 4 for 1,125 parameter sets. See *Massive parameter search* subsection in *Methods* section for more details on the definition of the score. Search of the other parameter set is shown in *Fig. S1*.

Results of Fig. 4 indicate that the normalized rate of TA formation 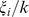 should be around 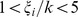 and the normalized rate of TA dissolution 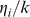 should be 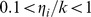 for 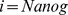 to reproduce the experimentally observed heterogeneous expression levels in ESCs. Here, the result of 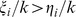 was needed to reproduce the observed feature that the HN peak height is larger than the LN peak height. Since the biologically reasonable lower bound of 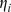 is the frequency of cell cycle 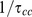, we use the lowest allowed value of 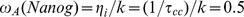 in the subsequent analyses by keeping 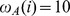 for 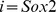, and *Oct4*. The precise values of other parameters are explained in *Parameters* subsection in *Methods* section. The simulated distributions of SON with this non-adiabatic switching of *Nanog* are shown in Figs. 3B and 3C. The simulated width of peaks is narrower than the observed one because in simulation the extrinsic noise due to the cell-cycle oscillation and the fluctuating reception of Lif are neglected for simplicity. The overall features of the distributions, however, agree well with the experimental data [14]: Nanog shows a clear two-peak distribution and the Oct4 distribution has an asymmetric single major peak.

**Figure 5:**
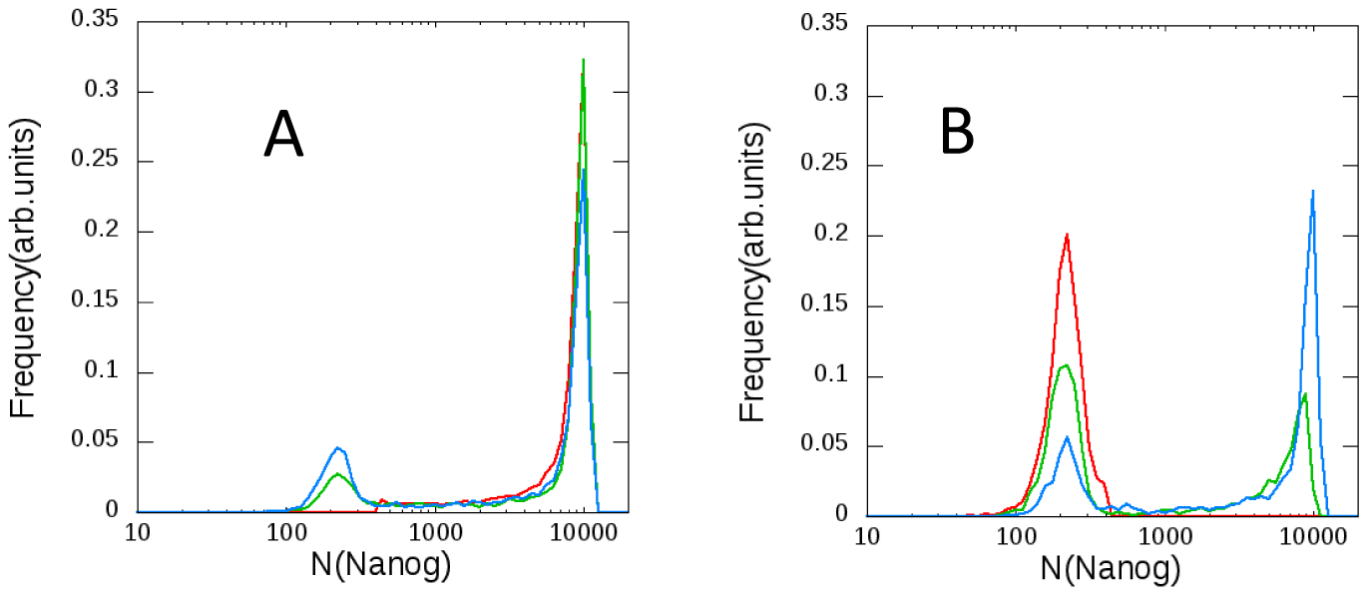
The simulated temporal change of distributions of the expression level of Nanog. Distributions starting from (*A*) the ensemble of 10,000 cells in the HN state, and (*B*) from those in the LN state. The distribution at the initial time (red), 1 day later (green), and 5 days later (blue). 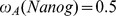 and 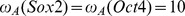.

Shown in Figs. 5A and 5B is the temporal change of distributions of Nanog calculated by starting from the ensemble of cells either in the HN or LN state at Day 0. Within several days, the single-peaked distribution of cells in either of the HN or LN state recovers the two-peak features, which reproduces the experimentally observed temporal relaxation [11], [14]. This relaxation indicates that ESCs show dynamical transitions between HN and LN states with timescale of a few days. The agreement between the observed and simulated timescales of transitions between HN and LN states indicates the validity of the small 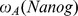 for the slow switching at the *Nanog* locus, and hence the data in Fig. 5 should rule out the other hypothetical models which can yield a bimodal Nanog distribution but with the large 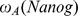.

A possible origin of the slow non-adiabatic switching of *Nanog* is the large scale chromatin reorganization in the formation/dissolution of TA of *Nanog*. This assumption of slow switching explains the observed two-peak distribution and the dynamical transition of the expression level of Nanog, and is also consistent with the single-peak distribution of Oct4. Thus, the assumption of the slow non-adiabatic switching of *Nanog* explains the observed phenotypic heterogeneity of ESCs.

### Diagram of transitions among cell states

Given the consistent model for the heterogeneity of ESCs, it is interesting to analyze how cells initiate differentiation. To simulate cells that can differentiate, the rate to turn the histone-code active, 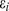, is increased to have a finite value for 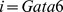 and 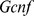. 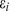 for 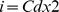 is also turned finite but kept small because in embryo, the distinct expression of Cdx2 is the event prior to the formation of inner cell mass from which ESCs are prepared, so that it is plausible to assume that the methylated DNA or the collective action of regulating factors inhibits the histone code of *Cdx2* from being active in ESCs (See subsection *Parameters* in *Methods*).

**Figure 6:**
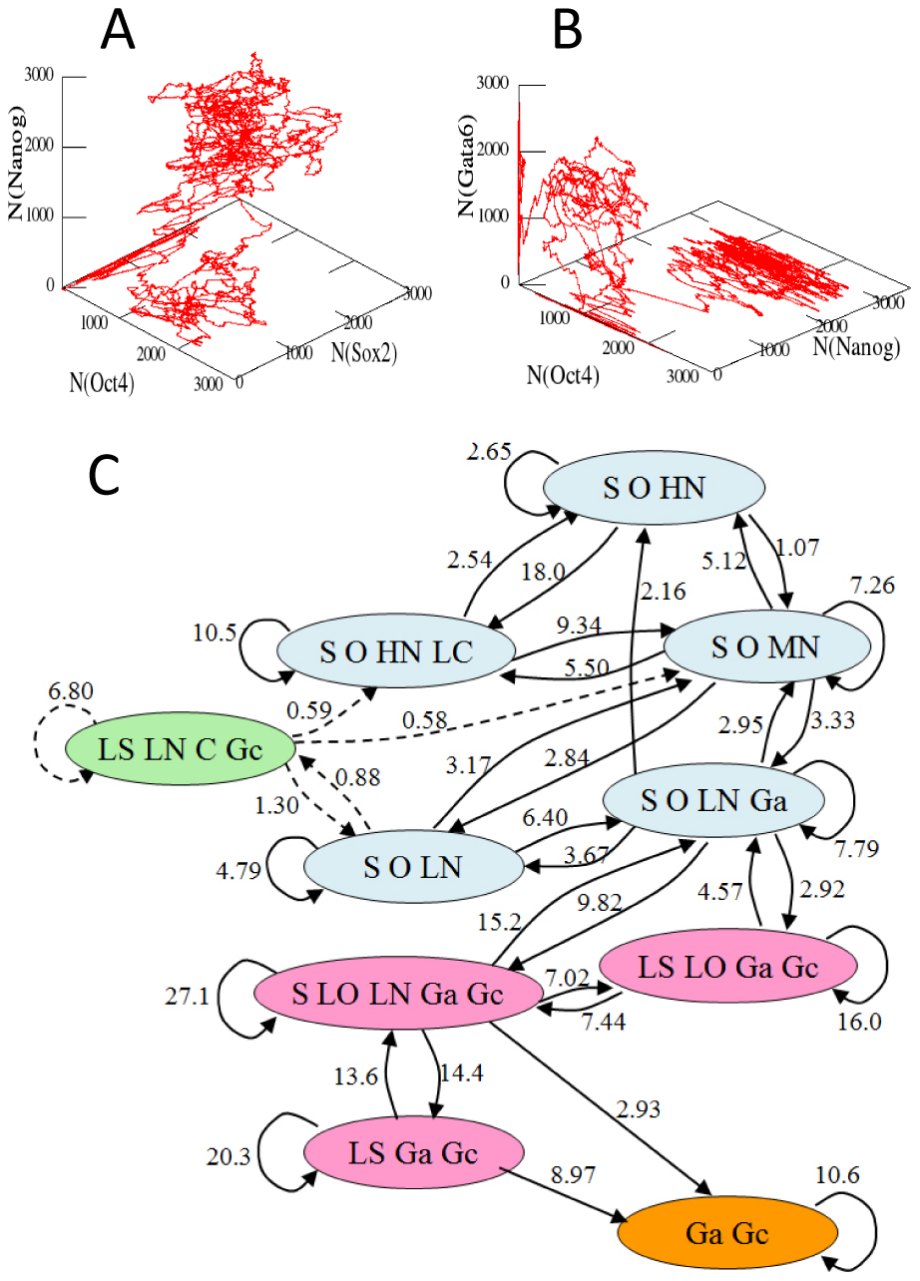
Simulated trajectory of differentiation can be regarded as a sequence of transitions. Example trajectories of 11.5 day length drawn with coordinates of (*A*) 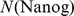, 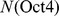, and 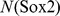, and (*B*) 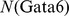, 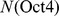, and 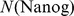. (*C*) Diagram of transitions among cell states. Cells wandering among ESC states (light blue) start differentiation through intermediate states (pink) or the Cdx2 expressing state (light green) to reach the Gata6 dominant state (orange), which leads to the primitive endoderm lineage. The transition rate 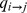 is written on the link connecting the 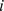
th to 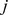
th cell states in units of 1/day. Arrows returning to the same states represent transitions via the infrequently appearing states which are not shown in the diagram according to the criterion described in the subsection *Transition diagram* in *Method* section. Each state is designated by the expressed factors; S (Sox2), LS (Low-level Sox2), O (Oct4) LO (Low-level Oct4), HN (High-level Nanog), MN(Middle-level Nanog), LN (Low-level Nanog), Ga (Gata6), Gc (Gcnf), and C (Cdx2). The diagram was obtained by sampling 10,000 cells simulated during 11.5 days. Transitions frequent more than the threshold defined in subsection *Transition diagram* in section *Methods* are extracted from trajectories. Transitions to/from the LS-LN-C-Gc state (dashed lines) were less frequent than the threshold but are drawn here to show the topological connectivity of the diagram.

Examples of trajectory simulated with this parametrization are shown in Figs. 6A and 6B. The trajectory in Fig. 6A wanders around several transient states but neither Cdx2 nor Gata6 dominates during this wandering: Cells are jumping among the states by maintaining the features of ESC. In Fig. 6B, on the other hand, the trajectory escapes from the ESC states to reach the Gata6 dominant state which is a gateway to the primitive endoderm lineage.

In both Figs. 6A and 6B, the trajectories are not the continuous drifts but consist of sojourns and jumps. This feature allows us to represent each trajectory as a sequence of transitions among “cell states”: Using the feature that the copy number of each factor, 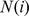, shows a multiple-peak distribution (Fig. S2), we divide each distribution into a few parts, each of which is named in an abbreviated way as HN (high-level Nanog), MN(middle-level Nanog), LN (low-level Nanog), S (high-level Sox2), LS (low-level Sox2), etc. The thresholds used to divide the distributions are summarized in Table 1. Then, cell states are defined by thus discretized distributions and also by a set of the histone states 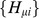. The trajectory is regarded as a sequence of transitions among those cell states. With this coarse-grained representation of trajectories, the mean waiting time for transition from the 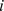
th to the 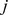
th cell states can be estimated as 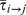, and the mean transition rate is defined by 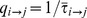 (See subsection *Transition diagram* in *Methods* for the detailed explanation on 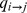).

**Table 1:** Definition of cell states and their labels.

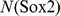	0–10	10–200	200 -	
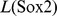 *	0	2	4	
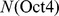	0–20	20–200	200 -	
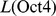	0	2	4	
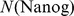	0–40	40–400	400–4000	4000 -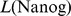	0	1	2	4
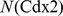	0–20	20–200	200 -	
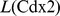	0	2	4	
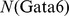	0–100	100 -		
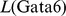	0	4		
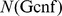	0–100	100 -		
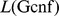	0	4		

The values of 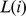 were determined for the visibility of Fig. 8.

In the case that the trajectory stays for a long duration at each cell state to erase its dynamical memory, this coarse-grained dynamics can be regarded as Markovian, or in other words, the transition probability from the 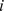
th to 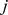
th states is not affected by which state the trajectory visited before reaching the 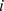
th state. It is suggested from Figs. 6A and 6B that the trajectories stay at each cell state long enough to show many oscillations during the stay, but the more quantitative test should be necessary to judge whether the coarse-grained dynamics is indeed Markovian or not. We leave this test as a future problem and proceed further in this paper to show how the transition diagram and the landscape view capture the important features of transitions among cell states.

Drawn in Fig. 6C is the diagram of transitions among thus defined cell states, where the value of 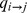 is shown on the link from the 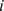
th to 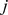
th cell states. In Fig. 6C the cell states in which all of Sox2, Oct4, and Nanog (SON) are expressed are regarded as the pluripotent states (or the ESC states) though the level of Nanog fluctuates largely among these states and sometimes Cdx2 or Gata6 coexists with SON. These ESC states are connected by loops of transitions and hence the cells wander among ESC states to wait for a chance to escape from the ESC states. Trajectories that have escaped from the ESC states go through the network of transitions among the intermediate states in which one or two of SON are lacking. From these intermediate states, cells reach the state in which Gata6 dominates. In some cell states, Cdx2 appears as fluctuation but the small value of 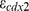 prevents Cdx2 from dominating the state.

It should be interesting to examine the validity of these predictions with the experimental observations: By quantifying the expression level of important factors, we will be able to define cell states from the experimental data. Then, we can check whether the differentiation is the process of jumping among these states. Though there is a global trend of kinetic flows from the ESC states to the differentiated states, the predicted pathways are not single but comprise the network of flows. It should be important to compare the predicted distribution of pathways as in Fig. 6C with the distribution of pathways experimentally observed by following the fate of individual cells in the culture.

### Epigenetic landscapes

**Figure 7:**
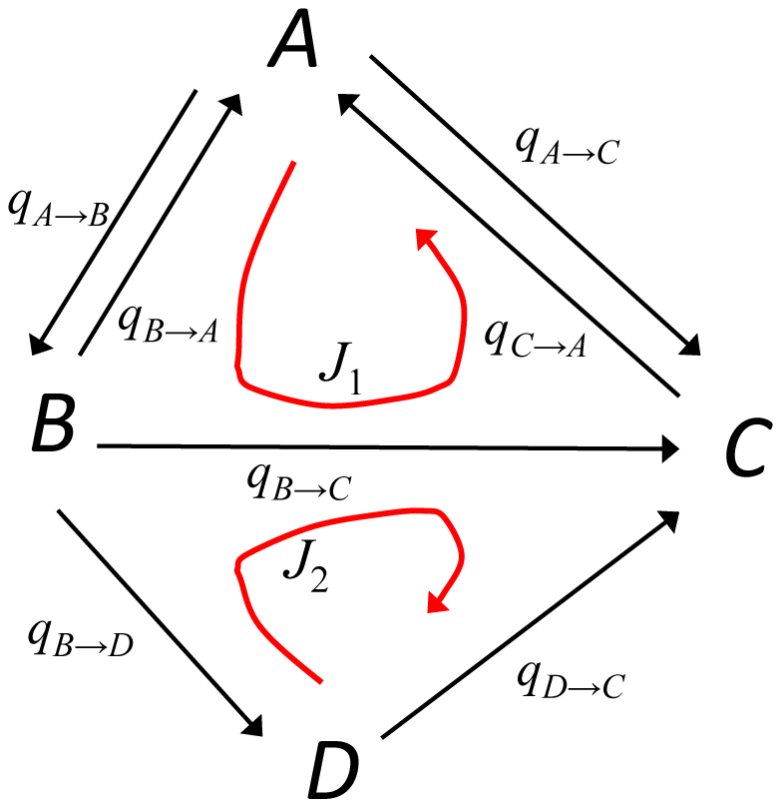
An example of looped diagram among cell states, 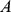, 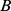, 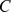, and 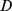. This diagram contains two loops. For two loops, two non-equilibrium curl fluxes, 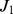 and 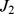, are introduced. Calculation of the epigenetic landscape and curl fluxes based on this diagram is explained in subsection *Epigenetic landscape* in section *Methods*.

To analyze dynamics of differentiation, the epigenetic landscape that underlies transitions among cell states should provide a useful perspective [63], [64]. Here, the landscape is derived from the transition diagram by using the analogy with the free energy surface in equilibrium dynamics. In equilibrium dynamics, by using the transition-state theory formula, the rate of transition from 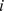 to 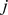
th states should be proportional to 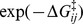 where 
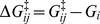
, and 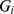 and 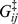 are the dimension-less free-energy like quantity at the 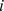
th state and at the transition state between the 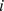 and 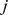
th states, respectively. We use this analogy to equilibrium dynamics by fitting the calculated rates 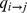 to this transition-state theory formula to obtain the free-energy like quantity 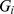 and 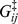. When the transition diagram has a tree-like structure without a loop, we can determine 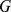 of each state one by one by fitting the simulated rates to this formula. We use this analogy with equilibrium dynamics as far as possible to draw the landscape 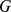 of non-equilibrium transitions. This method of fitting, however, apparently breaks down when the transition network contains one or more loops: When the transition network contains a loop, for example, we may attempt to determine 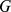 of states in the loop one by one by starting from the 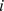
th state in the loop with the landscape value 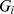, but at the end of traverse along the loop, we return to the initial 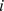
th state with a different value of 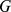 from the original 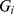. In this way, the fitting to the transition-state theory formula is inconsistent along loops. This inconsistency can be resolved when we explicitly consider the non-equilibrium feature of dynamics by introducing the curl flux of transition kinetics [65]–[68]. Thus, the kinetic process along each loop can be expressed by the combination of the landscape and a kinetic flow curling along the loop. Transitions, therefore, are described by the combined representation of landscape and non-equilibrium curl flux. An example of a looped diagram having curl fluxes is shown in Fig. 7. From 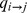 of this diagram, the free-energy like quantity 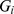 at the 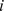
th cell state and 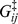 at the barrier between the 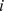 and 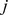
th cell states are calculated for 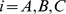, and 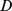, and curl fluxes 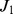 and 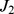 are obtained simultaneously. See subsection *Epigenetic landscape* in *Methods* for the explanation on how to calculate 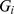, 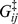, 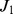 and 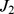 from 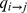 of Fig. 7.

**Figure 8:**
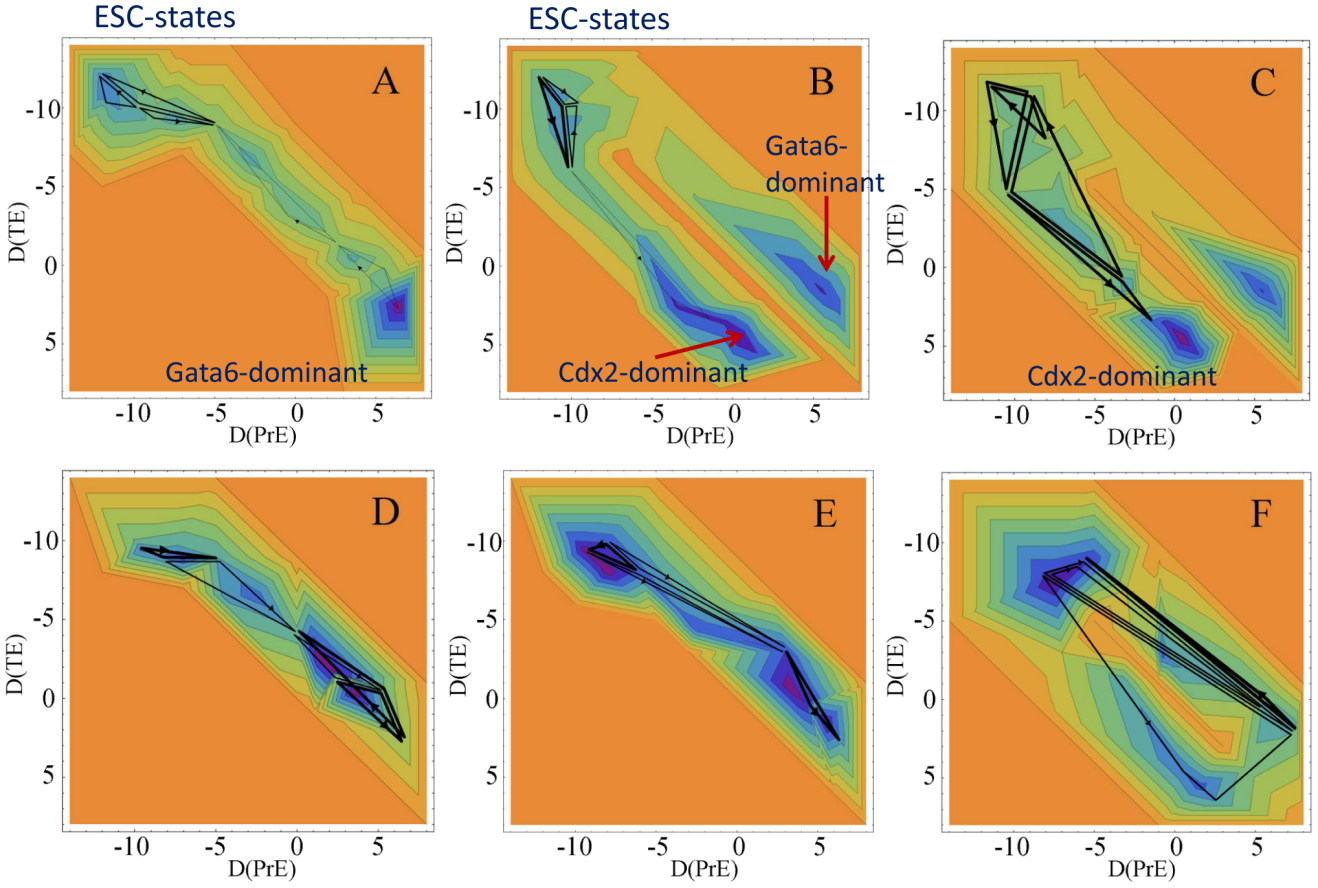
Epigenetic landscapes and non-equilibrium curl fluxes derived from the transition diagrams in the differentiation process. Contour maps of landscapes are shown on the two-dimensional plane of 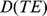, the degree of closeness to the trophectoderm lineage, and 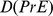, the degree of closeness to the primitive endoderm lineage, and curl fluxes are shown with black lines on the landscapes with the width of lines representing the strength of fluxes; thick lines (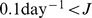), lines with the middle width (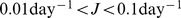), and thin lines (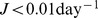). (*A*–*C*) For the slow *Nanog* switching with 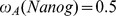 and 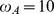 for other genes, and (*D*–*F*) for the fast *Nanog* switching with 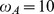 for all genes. The rate of Oct4 synthesis is (*A* and *D*) same as other factors, and (*B* and *E*) 25% and (*C* and *F*) 10% of other factors.

In Fig. 8 the landscapes and curl fluxes calculated from the simulated 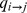 in the differentiation processes are shown on the two-dimensional plane with the coordinates of 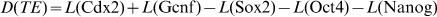 and 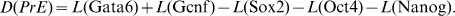. Here, 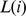 is the label of the discretized expression level of the 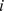
th factor, which is defined to have the larger value for the higher expression level. 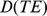 and 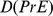, therefore, represent the degree of closeness to the trophectoderm and primitive endoderm lineages, respectively. The precise values of 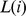 are chosen for obtaining good visibility of Fig. 8, and are explained in Table 1. In Fig. 8, the calculated 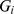 and 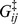 are plotted by assigning 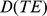 and 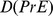 for 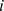 and 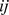, and 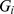 and 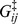 are interpolated by a smooth surface in the two-dimensional space of 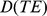 and 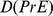.

The landscape corresponding to the diagram of Fig. 6C is shown in Fig. 8A. We see that the ESC states distribute on a flat basin in the region of small 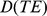 and 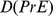: ESCs wander around this basin driven by both the fluctuations satisfying the balance between the forward and reverse transitions and the kinetic flow of curl flux that breaks the balance. ESCs start differentiation as they move along the valley stretching toward the Gata6 dominant state. Transitions among intermediate states along this valley are also accompanied by the weak non-equilibrium curl flux.

In Figs. 8B and 8C, the artificial depletion of Oct4 is simulated with the decreased rate of synthesis of Oct4. Since Oct4 and Cdx2 work in an antagonistic way, the depletion of Oct4 results in the stronger expression of Cdx2, which leads ESCs to the trophectoderm linage: With the decrease of the rate of Oct4 synthesis to 25% (Fig. 8B) and 10% (Fig. 8C) of the value in Fig. 8A, the landscape changes its shape by extending the valley toward the Cdx2 dominant state. In Fig. 8B two valleys to primitive endoderm and trophectoderm coexist with the curl flux on the basin of ESC states remaining, and in Fig. 8C the valley to trophectoderm dominates. These results are consistent with the experimentally observed induction of the trophectoderm lineage through the reduction of Oct4 [31].

Shown in Figs. 8D–8F are landscapes calculated with the assumption of the fast *Nanog* switching: 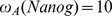. With this fast *Nanog* switching, the flat basin of the ESC states disappears, the curl flux in ESC states becomes localized, and ESCs quickly differentiate toward primitive endoderm (Fig. 8D). The curl flux on the ESC basin, therefore, originates from the slow *Nanog* switching. In other words, the eddy current associated with the non-adiabatic switching [23] manifests itself in the curl flux on the epigenetic landscape.

Difference between the slow and fast *Nanog* switching becomes more evident upon the reduction of Oct4 (Figs. 8E and 8F). With the fast *Nanog* switching, two valleys do not represent the distinct cell fate but they are directly connected to each other by the frequent transdifferentiation (Fig. 8F). This obscured differentiation arises from the averaged intermediate amount of Nanog synthesis under the fast *Nanog* switching. With the intermediate level of Nanog, the alleles of the lineage-specific genes tend to take the intermediate histone code as 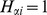 and 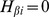 or 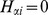 and 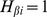. This intermediate level of activation of both *Cdx2* and *Gata6* increases the frequency of the transdifferentiation. With the slow *Nanog* switching, on the other hand, the histones of *Gata6* and *Cdx2* become either active with 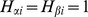 or repressive with 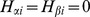, and such a clear-cut histone switching decreases the probability of the mixed expression of Cdx2 and Gata6. In this way the simulated results suggest that the distinct cell fate decision is based on the slow *Nanog* switching, so that the phenotypic heterogeneity of ESCs is necessary for the stable differentiation.

The present quantification of epigenetic landscapes showed that the model naturally reproduces the observed differentiation to primitive endoderm [10]. The model also reproduces the induced differentiation to trophectoderm observed when the Oct4 expression is artificially suppressed [3]. It should be interesting to further examine possibility of the predicted transdifferentiation due to the fast *Nanog* switching.

## Discussion

We developed a model of epigenetic dynamics and proposed a hypothesis that the timescale of formation/dissolution of TA decisively affects the self-renewal and differentiation of mouse ESCs. These effects can be checked experimentally by artificially varying the timescale of formation/dissolution of TA. The slower rate of formation/dissolution of TA for Oct4, for example, should give rise to the multi-peak distribution of Oct4, which should also affect the epigenetic landscape and non-equilibrium curl fluxes on the landscape. Further important is the application of the present ideas to engineering differentiation. Overexpression or repression of specific genes should alter the epigenetic landscape and curl fluxes, so that the calculation and observation of landscape and curl fluxes should provide a guideline for designing the process of cell differentiation.

An intriguing question is the effect of variation of the number of working alleles in a cell. In the present simulation, following the report for the single non-silenced *Nanog* allele in each ESC [51], only the single *Nanog* locus was considered in the simulation, which explained the bimodal Nanog distribution when the *Nanog* switching was slow. Assuming that both two alleles are working independently owing to the invalidated allelic regulation, we have three peaks in the Nanog distribution corresponding to the ‘high-high’, ‘high-low’ and ‘low-low’ levels of expression for two alleles of Nanog with the slow *Nanog* switching as shown in Fig. S3. This predicted three-peak distribution could be experimentally tested in ESCs, though the more careful investigation is needed on the possible correlation between the allelic regulation and the regulation of the timescale of gene switching.

The core part of the network relations among genes in the present paper was built from the experimental observations, but there are experimental suggestions still not taken into account in the present model. For example, a recent report suggested the auto-repression of Nanog [45]. This suggested interaction can affect the transition dynamics between the HN and LN states, which should be examined by simulation. The validity of the assumptions used in the present modeling of epigenetic dynamics should be checked by examining how the results are modified when the model is further extended. In the present model, three processes having the different timescales were considered; TF binding/unbinding, TA formation/dissolution, and the histone code modification. Each of these processes consists of multiple sub-processes, and therefore if the model is extended with the finer resolution, the involved timescales should have more variety [69]. The TA formation/dissolution, for example, may involve assembly of mediators and RNA polymerase, phosphorylation of these factors, chromatin looping, and the large scale change in the chromosome positioning in nucleus. In the present model, we treat them in a coarse-grained manner by representing the TA state with 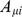 which takes a value between 0 and 1. By treating these multiple processes explicitly, we may be able to construct a more quantitative model that can be compared with experiments in more details, and the validity of the level of coarse-graining in the present model could be checked through such comparison.

We should stress, however, that the main conclusions on the importance of design of timescales of regulations and the usefulness of combined representation of landscape and non-equilibrium curl fluxes do not depend on the molecular details. Indeed, the simplified mathematical or statistical physical models to capture the essential features of landscapes and curl fluxes should be useful. The dynamical systems models, for example, emphasize the importance of oscillations in the gene network [70], which conforms with the view presented here on the importance of rotating curl fluxes.

Another important direction to improve the present model is to take into account the core genes that guide ESCs to primitive ectoderm which further differentiates into the primary germ layers. To develop the reliable models, the effects of cell-cell communication and cell cycles should be also taken into account. Especially, the cell-cell communication should play important roles to stabilize the cell type of colony of interacting cells [70], [71]. The model developed in the present paper was based on the assumption that the partial effect of cell-cell communication is implicitly taken into account by the mutual inhibition between Cdx2 and Gata6 (See *Methods* section). In order to analyze the differentiation process more quantitatively, the model needs to be extended to explicitly treat the effects of cell-cell communication. Those more elaborate models, together with the simplified statistical mechanical models, should reveal the rich phenomena in ESCs and differentiation processes.

## Methods

### Inference of the network from experimental observations

The model consists of interactions among six genes. Those interactions are inferred from the experimental data, which are complemented with various levels of assumptions as explained below. In the following, the assumptions used are categorized into Level A, Level B, Level C, and Others. The aim of the present study is not to claim the validity of those assumptions, but to clarify the mechanisms of epigenetic dynamics by using a set of biologically consistent assumptions. The interactions considered in Fig. 1 were inferred from the discussions below, which are numbered in the same way as interactions designated in Fig. 1:

**Level A**. Microarray or other genetic experimental techniques revealed the correlation or anti-correlation between expression levels of two genes, and the chromatin immunoprecipitation or other biochemical data showed the binding of one factor to the locus of the other gene. These data support the assumption that the transcription factor (TF) synthesized from one gene directly regulates the other gene. The Level A assumptions give the backbone of the present model of the regulatory network.

1. Each of *Oct4*, *Sox2* and *Nanog* loci has the Oct/Sox enhancer region [7], [72], on which Oct4 and Sox2 bind together to form the Oct4-Sox2 complex to activate *Oct4*, *Sox2*, or *Nanog* [4], [7], [72]. There are two possible ways of binding though they are not mutually exclusive; The Oct4-Sox2 complex is formed *before* they bind to DNA, or Oct4 and Sox2 bind to the adjacent sites of DNA to form the complex *after* binding. These two ways of binding are different in their cooperativity in the binding process. However, since the cooperativity of binding is masked by the cooperative formation/dissolution of transcription apparatus (TA) in the present model, these two ways of binding do not give significant difference in the switching behavior. We use, for simplicity, the latter assumption of forming complex *after* binding to DNA, but represent the effects of complex formation by assuming that the binding of either one of Oct4 or Sox2 is not enough but the binding of both two factors are needed for forming TA (We assume that the formation of the Oct4-Nanog complex is another route to form TA).

2. Gcnf binds to the *Oct4* and *Nanog* loci to repress them [48].

3. The Oct4-Cdx2 complex represses both *Oct4* and *Cdx2* [47], [73].

4. Nanog binds directly to the *Gata6* locus to repress it [13].

5. Because the binding of Oct4 to the *Nanog* locus is necessary for forming the higher order structure of chromatin at the *Nanog* locus [61] and the binding site of Oct4 is adjacent to the binding site of Nanog at the *Nanog* locus [6], we expect that the Oct4-Nanog complex formed on the chromatin is necessary for building the TA of *Nanog*.

6. Nanog promotes the expression of Oct4 [5] and both Nanog and Oct4 directly bind to the *Oct4* locus [6]. Because the binding site of Oct4 is in proximity of the binding site of Nanog at the *Oct4* locus [6], we assume the promotion of the formation of TA of Oct4 through the binding of Oct4-Nanog complex on the *Oct4* locus.

7. Nanog is suggested to promote expression of Sox2 [8], [74] and both Nanog and Oct4 directly bind to the *Sox2* locus [6]. Because the binding site of Oct4 is in proximity of the binding site of Nanog at the *Sox2* locus [6], we assume that the formation of TA of Sox2 is promoted by the binding of Oct4-Nanog complex on the *Sox2* locus.

**Level B**. Genetic experimental data showed the correlation or anti-correlation between expression levels of two genes, but the direct evidence for the physical interactions between two genes are not yet obtained. In this case, the interactions can be indirect through the other unidentified factors. Even in that case, we may assume the hypothetical direct interaction between two genes in the model. Such assumption is reasonable in the coarse-grained model, in which the multiple detailed molecular processes are summarized into one process.

8. Excess expression of Oct4 reduces the expression level of Nanog [75]. We assume in the model that the *Nanog* locus has multiple binding sites of Oct4 and the occupation of the part of those sites is necessary for the formation of TA, but the occupation of all sites increases the rate for making the histone-code repressive.

9. The Nanog-null ESCs differentiate into cells similar to those induced by Gata6-positive cells [2], [76]. Since Gata6 and Nanog work in an antagonistic way [73], [77], we assume that Gata6 and Nanog are mutual repressors. Though it is not clear whether the repression of Nanog by Gata6 is direct or indirect through the other factors, we represent the interaction as a direct one by following the spirit of the coarse-grained approximation.

10. *Gcnf* is positively regulated by Gata6 and Cdx2 [10]. We assume for simplicity that *Gcnf* is activated directly by Gata6 and Cdx2 in the model.

**Level C**. No precise genetic data is available on the correlation or anti-correlation of gene activities, but from functional or biological observations, it is reasonable to assume the relation between two genes. The assumed interaction on such phenomenological basis might be a summary of the action of the larger network, but its representation as a single hypothetical process is useful to make the model behavior biologically reasonable.

11. Upon the removal of Lif or other agents from the culture, ESCs start differentiation. Then, each cluster of differentiated cells do not return to ESCs spontaneously. This stabilization of the differentiated cells may be enhanced by the positive feedback among the lineage-specific genes as was assumed by [63]. This effect of the regulatory network is represented in the model as auto-activation of lineage-specific genes, *Gata6*, *Cdx2* and *Gcnf*. These auto-activating interactions may be phenomenological or hypothetical interactions.

12. The cluster of cells differentiated into one lineage do not spontaneously transdifferentiate into the other lineages. This inhibition of transdifferentiation may arise from the reception of the external factor that is secreted or exhibited by the neighbor cells in the cluster. Such effect of the cell-cell communication is phenomenologically represented in the model by the mutual repression of genes specific to the different lineages.

**Others**. Other biochemical or biophysical data showed the existence of interactions.

13. Nanog dimerization is essential for the self-renewal of ESCs [19]. Nanog dimerization can be the faster process than its binding to the loci, so that we assume in the model that all the interactions between Nanog and chromatins are through the dimerized Nanog.

As assumed in the above argument, each locus of gene has multiple binding sites of TFs. From Fig. 1 we define the binding sites in the model as in Table 2.

**Table 2:** The binding sites of TFs on each locus of gene in the model.

locus	activator binding site			repressor binding site		
	1	2	3	1	2	3
*Sox2*	Sox2	Oct4	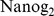	-	-	-
*Oct4*	Sox2	Oct4	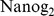	Cdx2	Gcnf	-
*Nanog*	Sox2	Oct4	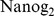	Oct4*	Gata6	Gcnf
*Cdx2*	Cdx2	-	-	Oct4	Gata6	-
*Gata6*	Gata6	-	-	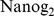	Cdx2	-
*Gcnf*	Cdx2	Gata6	Gcnf	-	-	-

The secondary Oct4 bound on the *Nanog* locus works in repressive ways only in the reactions of Eqs15 and 16.

### Reactions in the model

The state of the allele 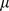 of gene 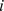 in the model is represented by variables 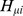, 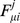, and 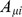, where 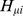 represents whether the histone code is active (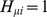) or repressive (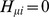), 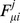 represents whether the 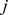
th TF is bound (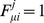) or unbound (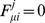), and 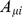 represents whether the TA of the locus 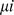 is formed and ready for transcription (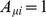) or unformed and not ready (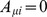). TA may be partially formed when the incomplete number of TFs are bound on a locus, and hence we write 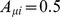 to represent such a partially formed TA. The copy number of the 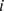
th protein is represented by 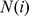. The temporal changes of 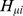, 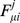, 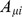, and 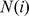 are numerically followed by using the Gillespie algorithm [58], which simulates the reactions explained below.

The 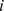
th protein is synthesized from the locus 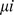 in a burst-like fashion with the rate 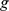 as
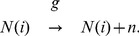
(1)Here, we assume that the burst size 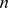 stochastically fluctuates in each burst with the probability of the distribution, 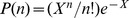, with 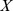 being the average burst size. Though the distribution of the burst size was reported to obey the geometric distribution in bacteria [78], the precise distribution of the burst size in higher organisms is not known [79]. We here used the Poisson distribution to highlight the effects of the burst size, but as shown in Fig. S1, the model behavior does not sensitively depend on the burst size 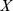, and hence we expect that the difference in distribution does not much affect the results. The 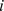
th protein is degraded with the rate 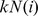 as
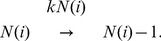
(2)From Eqs.1 and 2, we can see that the representative copy number of each protein is 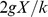, where the factor 2 comes from two available alleles. The synthesized 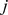 th protein can bind to and unbind from the 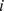 th site if the locus 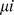 has the binding site for the 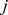 th protein (Table 2). The binding rate, 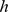, depends on the copy number of the protein to be bound. We assume the simplest linear relation by introducing a constant 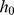 as 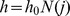. When the TF cooperatively binds in a form of oligomer, the contribution of higher orders of 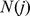 should be taken into account in 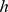. However, in the present model, unlike the bacterial cases, the modification of 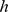 with the higher order terms of 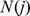 does not affect the model behaviors significantly because the cooperativity of switching is dominated by the formation/dissolution of TAs. We therefore assume
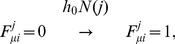
(3)

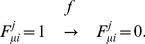
(4)We should note that for the frequent switching between 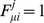 and 0 to take place, the ratio 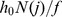 should be around 1, or 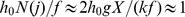. For 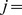
Nanog, dimerization should be much faster than other processes, so that we use the copy number of Nanog dimer, 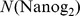, as 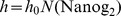 in Eq.3 instead of the total copy number of Nanog, 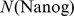. 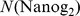 can be estimated from the equilibrium relation as
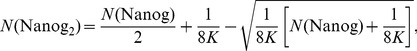
(5)where 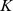 is the equilibrium constant of dimerization. In Eq.2, the copy number of the monomeric Nanog, 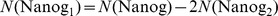 is used to define the degradation rate as 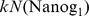.

The binding of activator TFs triggers the formation of TA; starting from 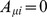 through the intermediate state 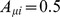 to reach 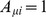 as
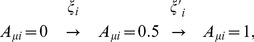
(6)and the TA is resolved stochastically as
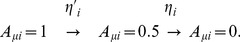
(7)These formation/dissolution of TA should be largely affected by the state of histones at that locus. The change of the histone code is simulated by switching between the active and repressive states as
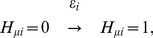
(8)

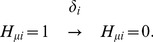
(9)In Eqs.6–9 the rates 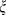, 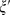, 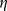, 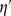, 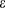, and 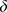 are represented with a suffix 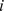 to emphasize their dependences on the type of gene.

We next explain how the rates defined in Eqs.1–9 depend on the gene state, 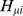, 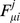, and 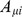. 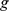 in Eq.1 depends on whether the TA is formed or not, which is represented by the variable 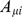. By using constants 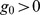 and 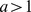, we write 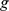 as
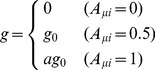
(10)Notice that with this rule the protein is synthesized only when the TA is formed at least partially as 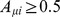.

The rates of TA formation and dissolution depend on how TFs are bound on the locus. Since TFs can be either of repressor or activator when they bind on the particular locus, we distinguish the binding sites 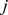 by writing 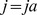 when the 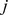 th TF is an activator of 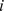, and 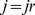 when the 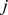
th TF is a repressor of 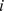. With this representation, the rate for the first step of TA forming, 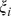 in Eq.6, is represented as follows;
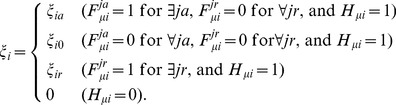
(11)Notice that the TA is formed with 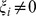 only when the histone code is active (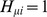). Here, 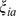 is the rate when some activator TF is bound on the locus but there is no bound repressor, 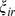 is the rate when some repressor is bound on the locus, and 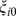 is the rate when no activator or repressor in the model is bound on the locus. Even in the case there is no bound activator in the model, other TFs which are not represented in the model may bind on the locus to promote the TA forming, and hence we assume that the basal background rate for the TA formation, 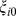, is finite. Considering these definitions, we expect 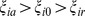. For the second step of TA forming, we expect that all possible TFs should bind on the locus to complete the TA formation, so that we have
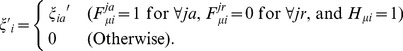
(12)For *Gcnf* and *Sox2*, we do not consider the repressor explicitly, so the rule is simplified as
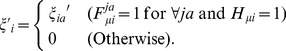
(13)


The rates of the histone-code modification, 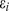 in Eq.8 and 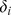 in Eq.9, should also depend on the state of gene. We assume that the histone code can be turned active only when enzymes to modify the histone code are recruited by activators and are not inhibited by repressors. Therefore, 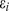 becomes 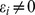 when the gene state is similar to the situation for 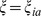, i.e., when some activator TF binds and no repressor binds on the locus;
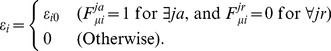
(14)For 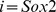, *Oct4* and *Nanog*, the histone-code activation is promoted by binding the Oct4-Sox2 or Oct4-Nanog complex. To represent cooperativity due to this complex formation, we modify the rule of Eq.14 as
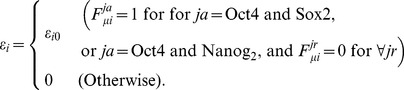
(15)We assume that 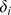 becomes 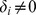 in the opposite situation, i.e., when some repressor binds and no activator binds on the locus. For all genes except Gcnf and *Sox2*, we have
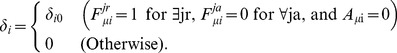
(16)No repressor is assumed to bind on the *Gcnf* or *Sox2* locus in the model, so that for 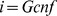 or *Sox2* we have the rule,
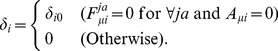
(17)


Eqs.1–9 were simulated with the Gillespie algorithm with the rates defined by Eqs.10–17. The simulation was started from the following initial condition which represents the pluripotent state,
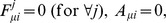


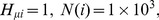
(18)for 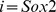, *Oct4*, and *Nanog* and
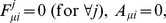


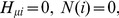
(19)for 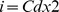, *Gata6*, and *Gcnf*. Starting from this initial condition, the simulation for 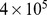
sec was performed by keeping 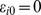 for 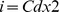, *Gata6*, and *Gcnf* to reach the steady ESC state. This first 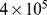
sec trajectory was discarded and the data were sampled after that for the statistical analysis by keeping 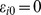 for 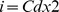, *Gata6*, and *Gcnf* to simulate ESCs or by turning 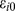 to be 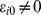 for 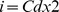, *Gata6*, and *Gcnf* to simulate the differentiation process.

### Parameters

The model has parameters, 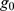, 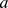, and 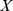 for protein synthesis, 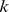 for protein degradation, 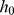 and 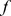 for binding and unbinding of TF, 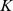 for Nanog dimerization, 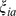, 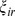, 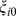, 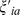, 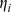 and 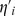 for formation and dissolution of TA, and 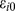 and 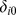 for the histone-code modification. For simplicity of description, the suffix 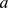 of 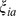 and 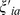, and the suffix 0 of 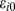 and 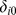 are suppressed in the previous sections of *Model* and *Results*, in Fig. 2, and in Table 3. Parameters were determined according to the following guideline: (1) Parameter values were not tuned in a precise way but only their orders of magnitude were taken care of. (2) The same parameter was set to have the same value for different genes as far as possible. In the following, in order to determine the ranges that parameters can take with this guideline, we first discuss two basic quantities, the period of cell cycle 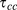 in the following items 1 and 2, and the typical copy number of each protein in a cell in the item 3.

**Table 3:** Order of magnitude of parameters in the model.

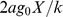 *	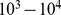	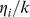	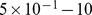
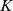	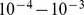	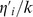	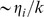
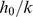	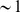	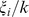	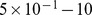
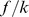	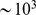	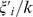	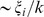
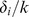 †	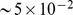		
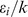	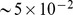		

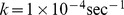
.

Subscript 0 or 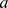 in 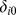, 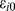, 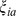, and 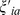 is omitted for simplicity.

1. A basic timescale for the description of cellular processes is the period of cell cycle. The period of cell cycle of ESCs is 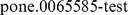
sec 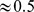
day, which is shorter than that of somatic cells. Though the periodic modulation of cells along the cell cycle is not explicitly treated in the present paper, 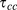 can be used as a measure of timescales of other processes. The timescale of binding/unbinding of TF should be much shorter than 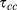, or in other words, the rates of binding/unbinding of TF should be much larger than 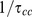. The timescale of TA formation/dissolution should not exceed the cell cycle period, so that the rates of TA formation/dissolution should be similar to or larger than 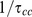. Since the histone code is often inherited across the cell cycle [35], the timescale of the histone-code modification should be larger than 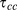, or in other words, the rates of histone-code modification should be smaller than 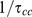.
2. Though the cell cycle period is a convenient measure of biological processes, it is not explicitly used in the model. Alternatively, we use the lifetime of proteins, or the rate constant of protein degradation 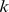, as an explicit measure of processes in the model. From the observed variation of the copy number of Oct4 [4] and Nanog [16], we have 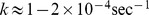, which is larger than the frequency 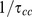 of cell division of ESCs; 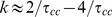. In the following, instead of 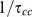, we use 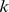 as a measure to quantify the other parameters and to define adiabaticity.
3. Another important quantity is the typical copy number of proteins in a cell. It has been observed that this copy number is 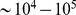 [80], which is much larger than that in bacteria (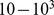) [81] due to the much larger cell volume and the larger number of working ribosomes in eukaryotic cells. The copy number of TFs which concentrate in nucleus, however, should be smaller than that of proteins working in cytosol because the volume of nucleus is about 10% of the whole cell volume. Therefore, by assuming that the similar concentration to the one of cytosolic proteins is transported and accumulated in the nucleus, the typical value of the copy number of TFs should have the range of 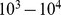. Therefore, we should have 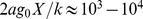.
4. The rate of TF binding/unbinding may not be much different from that in bacteria, so that 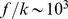. For the sensitive gene switching, the probability that a TF binds to the locus should not be so close to 1 or 0, so that 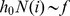. Given 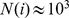, we have 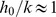.
5. The Nanog dimerization constant is 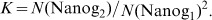. By assuming 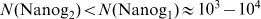, we have 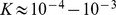.
6. The rate to change the chemical state of a nucleosome may be as large as 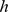 or 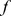 because each reaction to add (remove) the methyl or acetyl group to (from) a nucleosome should be catalyzed by enzymes recruited by TFs [9], [10]. The chemical state of an array of nucleosomes along each of gene loci, however, is changed in a cooperative way and should show the much longer lifetime. Indeed, the chemical state of nucleosomes, i.e., the histone code is often inherited across generations of cell cycle, and it has been observed that the timescale to change the histone code of *Oct4* in ESCs is several days, which is approximately 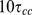 [35]. We assume, therefore, the rate to change the histone code to be 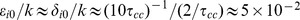.
7. The timescale of TA formation/dissolution is not known, but we assume it to be slower than TF binding/unbinding and faster than the cell cycle. The latter assumption of the larger rate of TA formation/dissolution than the cell-cycle frequency should be reasonable because the TA structure should be reset and resolved in the large scale reorganisation of chromatin structure at the time of cell division. The former assumption of the slower rate than the TF binding/unbinding is also reasonable when we consider that TA formation is the much more complex process including assembly of many factors, looping of chromatin, and chemical modification of RNA polymerase. Therefore, we examined the wide range of 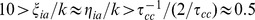. The completion of TA may be the similar process to the initiation of the formation of TA, so that we assume 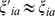 and 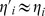. Other conditional values of 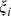 are defined in Eq.11 as 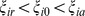.

From the above consideration, we can estimate the orders of magnitude of parameters, which are summarized in Table 3. In order to further analyze the meaning of difference in the order of magnitude of these parameters, we define the dimension-less parameters, adiabaticities, as measures of relative rates of individual processes to the rate of the protein copy-number change: We have three adiabaticities in our model of epigenetic dynamics, 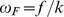 which measures the relative frequency of the TF binding/unbinding, 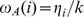 which measures the relative frequency of the TA formation/dissolution, and 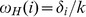 which measures the relative frequency of the histone-code modification. From the above estimation, the orders of magnitude of adiabaticity are 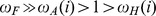 or 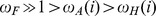. Therefore, the TF binding/unbinding is strongly adiabatic, and the histone-code modification is non-adiabatic. The TA formation/dissolution is adiabatic or non-adiabatic depending on the type of gene, which characterises the dynamic behavior of the present model.

The parameters used in Figs. 3, 5, 6, and 8 in *Results* section are shown in Table 4. We can see that the values of Table 4 are within the range shown in Table 3. In Table 4, the dependence of parameters on the type of genes is minimized: The specific values deviating from the most common values are 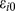 for 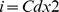, and 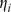, 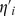, 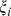, and 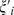 for 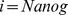. Since the differentiation to trophectoderm takes place prior to the formation of inner cell mas (ICM) in the early embryo and ESCs are prepared from ICM, it is reasonable to assume that the differentiation to trophectoderm is somehow suppressed in ESCs. We express this tendency by using the smaller value of 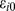 for 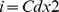, the marker protein for trophectoderm. The small value of 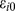 for 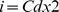 represents the possible inherent effects of silencing *Cdx2* in ESCs.

**Table 4:** The parameter set used in Figs. 3, 5, 6, and 8 in the section *Results*.

	*Sox2*	*Oct4*	*Nanog*	*Cdx2*	*Gata6*	*Gcnf*
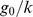 *	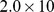					
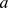	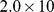					
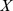	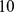					
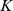	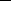		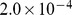	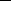		
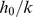	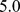					
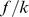	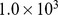					
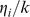	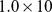		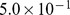 ‡	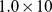		
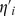 /k	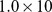		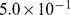 ‡	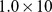		
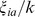	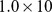		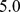 ‡	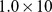		
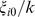	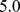		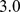 ‡	5.0		
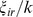	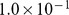					
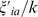	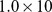		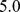 ‡	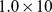		
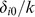	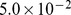					
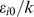	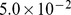			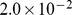 †	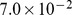 †	

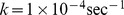
.

These values are turned to 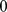 to simulate ESCs cultivated in media that can keep cells in the self-renewing pluripotent state.

Simulations with the fast *Nanog* switching were performed by making these values identical to the values for *Sox2* or *Oct4*.

The smaller values of 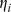, 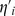, 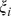, and 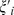 for 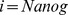 are the manifestation of the slow switching dynamics of the *Nanog* locus, which is a main feature of the present model of epigenetic dynamics as explained in Figs. 3B, 3C, 5, 6, and 8A–8C. To clarify the effects of this slow switching, we also calculate for comparison by using the same values of 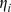, 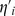, 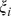, and 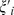 for 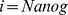 as those for *Sox2* or *Oct4* as shown in Figs. 3A, 3C, and 8D–8F.

### Massive parameter search

From Table 3, the order of magnitude of most of parameters are determined and their typical example values can be adopted as in Table 4. In Table 3, however, values of some parameters are undetermined yet. We perform a massive parameter search for values of these parameters to find the consistent values with the experimentally observed results.

An important undetermined set of parameters are 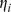 and 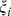. We adopt the values in Table 4 for other parameters, and as discussed in *Parameters* subsection, we impose the constraints 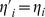 and 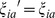. Considering the constraint of 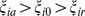, we assume a small value for 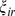 as in Table 4 and also assume 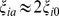. Then, the parameters 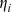 and 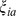 are left undetermined. In Fis.3A, 3C, and 8D–8F, we used the values 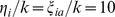 to represent the situation that the TA formation/dissolution is much slower than the TF binding/unbinding but faster than the protein copy-number change, i.e., 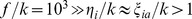. This choice of values for 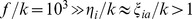 and 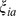, however, is not consistent with the experimentally observed distributions of expression level of SON as is shown in Fig. 3, and hence we examined the other values of 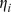 and 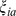 for 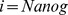.

We generated about 1,000 parameter sets scattered on the two-dimensional plane of 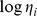 and 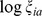. For each of these generated parameter sets, we calculated 10,000 trajectories and obtain the distributions of expression level of SON by averaging over the trajectories. We then evaluated the score 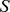 as
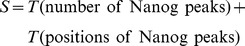


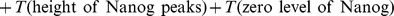


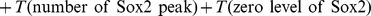


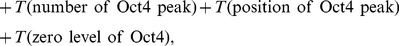
(20)where each term of Eq.20 is 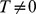 when the simulated data agrees with the corresponding feature of the observed data as shown in Fig. 3D, and 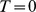 otherwise:

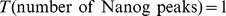
 when the simulated distribution of expression level of Nanog has two peaks (HN and LN peaks).

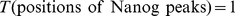
 when the ratio of the expression level at the HN peak to that at the LN peak, 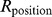, is 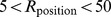.

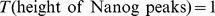
 when the ratio of the height of the HN peak to that of the LN peak, 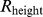, is 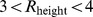 or 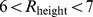, and 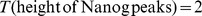 when 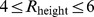.

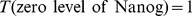
 when the population at the zero expression of Nanog is less than 2%.

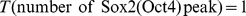
 when the distribution of the expression level of Sox2 (Oct4) is single peaked.

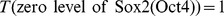
 when the population of zero expression of Sox2 (Oct4) is less than 2%.

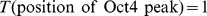
 when the expression level at the peak of Oct4 distribution is in between expression levels at the HN and LN peaks.

In this way, 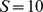 when all the observed features of the distributions of expression level of SON in ESCs are reproduced by the simulated data. In Fig. 4, 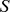 for 1,125 parameter sets is plotted on the plane of 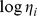 and 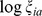 with 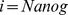.

The other undetermined set of parameters in Table 3 are the bare rate of protein synthesis, 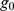, the ratio of the rate of synthesis at the completed TA to that of the partially formed TA, 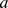, and the average burst size, 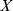. Since we have a constraint 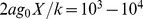 as in Table 3, we fixed 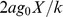 to the value 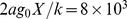 as in Table 4 and searched the values of 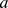 and 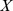 extensively by modifying 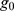 according to the constraint 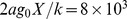. 1,125 parameter sets were generated as scattered points on the two-dimensional plane of 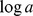 and 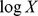 and 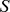 were calculated by averaging over 10,000 trajectories for each of the parameter sets. The calculated 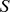 is plotted in *Fig. S1*, which shows that 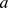 should be within the range of 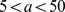 especially to satisfy 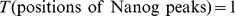 and the results are not sensitively dependent on the burst size 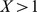.

### Transition diagram

The total time length, 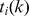, during which the 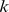
th trajectory stayed at a certain cell state 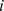 is calculated. By sampling 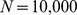 trajectories of 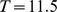 days, the averaged frequency of the appearance of the state 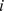, 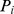, is obtained as 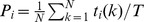. We can see in *Fig. S4* that the small number of states appear much more frequently than the other many states. We disregard the rarely appearing states and draw the transition diagrams among the states whose 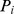 are larger than a threshold value 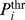. Here, we choose different thresholds for different transition diagrams because the difficulty to solve simultaneous equations for landscape and fluxes depends on the topology of the diagram. The larger threshold makes the diagram simpler to increase the solvability of equations, but we use the smallest possible threshold; 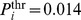 for Fig. 8A, 0.016 for Figs. 8B–8E, and 0.029 for Fig. 8F.

Then, the time of the trajectory needed for the transition from the 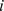 th to 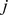 th states is monitored and recorded as 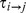. 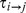 is averaged along the trajectory and over the ensemble of trajectories to obtain 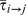. The transition rate is defined by 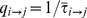. The link 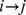 between two cell states 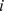 and 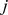 is drawn in the transition diagram when the transition 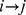 is observed more frequently than the threshold times, which are 200 (Fig. 8F), 600 (Figs. 6C, 8A, 8B, and 8E), 700 (Fig. 8C), or 900 (Fig. 8D) times in the sampled ensemble of trajectories. The transition diagram of Fig. 6C is drawn by connecting the cell states with thus defined links of transitions.

### Epigenetic landscape

The epigenetic landscape, 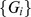 with 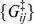, is calculated with the following rules:

- Given 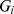 at the 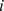
th state, 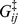 at the saddle between the 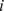
th and 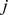
th states is calculated by fitting the expression 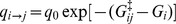 with 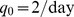 to the simulated rate by regarding 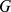 as a quantity analogous to free energy.
- After obtaining 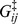, 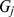 is calculated by fitting the expression 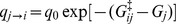 to the simulated rate of the reverse transition.
- When the reverse transition does not appear in the simulated trajectories, a threshold value 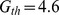 (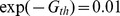) is used to represent the high enough barrier to inhibit the reverse transition within the simulated timescale. We then have 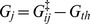.
- The above three rules can not be applied for the looped part in the transition diagram. This problem is solved by combining landscape and curl flux to represent the kinetic flow on the landscape.

The last rule can be explained by using an example of Fig. 7, in which four cell states 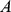, 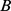, 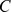, and 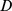 are connected by directed links that represent transitions among cell states. For the diagram of Fig. 7, we should solve the following equations:
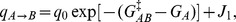


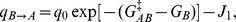


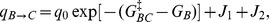


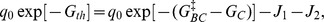


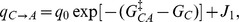


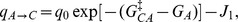


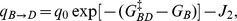


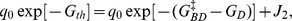


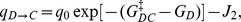


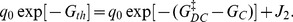
(21)These equations have 11 variables. When we fix 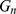 at a certain state 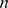, the relative height 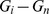 at other three states, 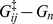 at five saddles, and two currents 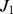 and 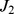 are obtained by solving the above 10 equations simultaneously.

## Supporting Information

Figure S1
**Search for a range of parameter set** 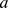 **and** 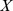 **.** The ability of the simulated trajectories to reproduce the experimentally observed data of distributions of the expression level of SON is evaluated by the score 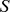 which is defined in Eq.20 in *Methods* section. The score was evaluated for each of 1,125 parameter sets scattered on the two-dimensional plane of 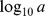 and 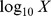, where 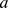 is the ratio of the rate of protein synthesis from the fully formed TA and that from the partially formed TA, and 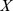 is the average burst size.(TIF)

Figure S2 **Simulated distributions of the copy number of protein factors which appeared in trajectories of the differentiation process from the ESC states to the Gata6-dominant state.** Distributions are divided to define the cell states by introducing thresholds designated by arrows. The abbreviations used to refer the cell states in Fig. 6C of the main text are written on each panel. 10,000 trajectories for 11.5 days were used for sampling the data.
(TIF)

Figure S3
**Distribution of expression level of Nanog in the case that either of two alleles of** *Nanog* **is not silenced through the allelic regulation.** (A) 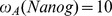 and (B) 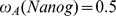. Other parameters are the same as those used in Fig. 3 of the main text.(TIF)

Figure S4
**Probability** 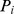 **of the appearance of the cell state** 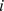 **in the 10,000 simulated trajectories are shown in the rank order of** 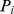 **.**
(TIF)
