# Supplementary figures and images for: Time Scales in Epigenetic Dynamics and Phenotypic Heterogeneity of Embryonic Stem Cells
Source: PLoS Comput Biol. 2013 Dec 12;9(12):e1003380. doi: 10.1371/journal.pcbi.1003380 (PMC3861442; doi:10.1371/journal.pcbi.1003380)

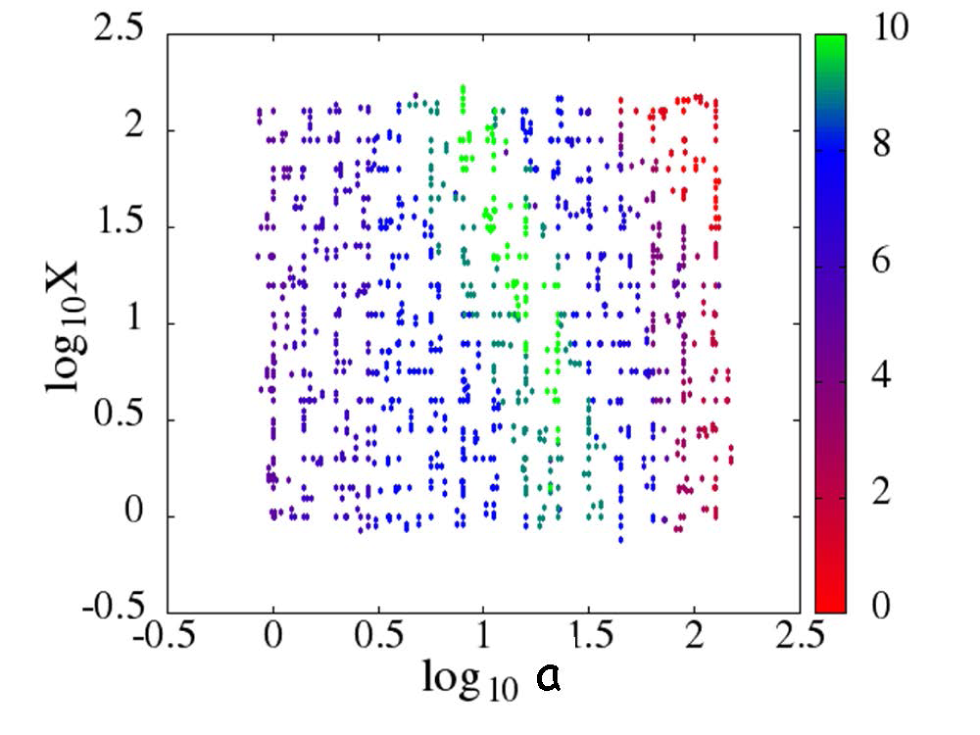

Supplement: Figure S1 — Search for a range of parameter set and . The ability of the simulated trajectories to reproduce the experimentally observed data of distributions of the expression level of SON is evaluated by the score which is defined in Eq.20 in Methods section. The score was evaluated for each of 1,125 parameter sets scattered on the two-dimensional plane of and , where is the ratio of the rate of protein synthesis from the fully formed TA and that from the partially formed TA, and is the average burst size. (TIF) [file pcbi.1003380.s001.tif]

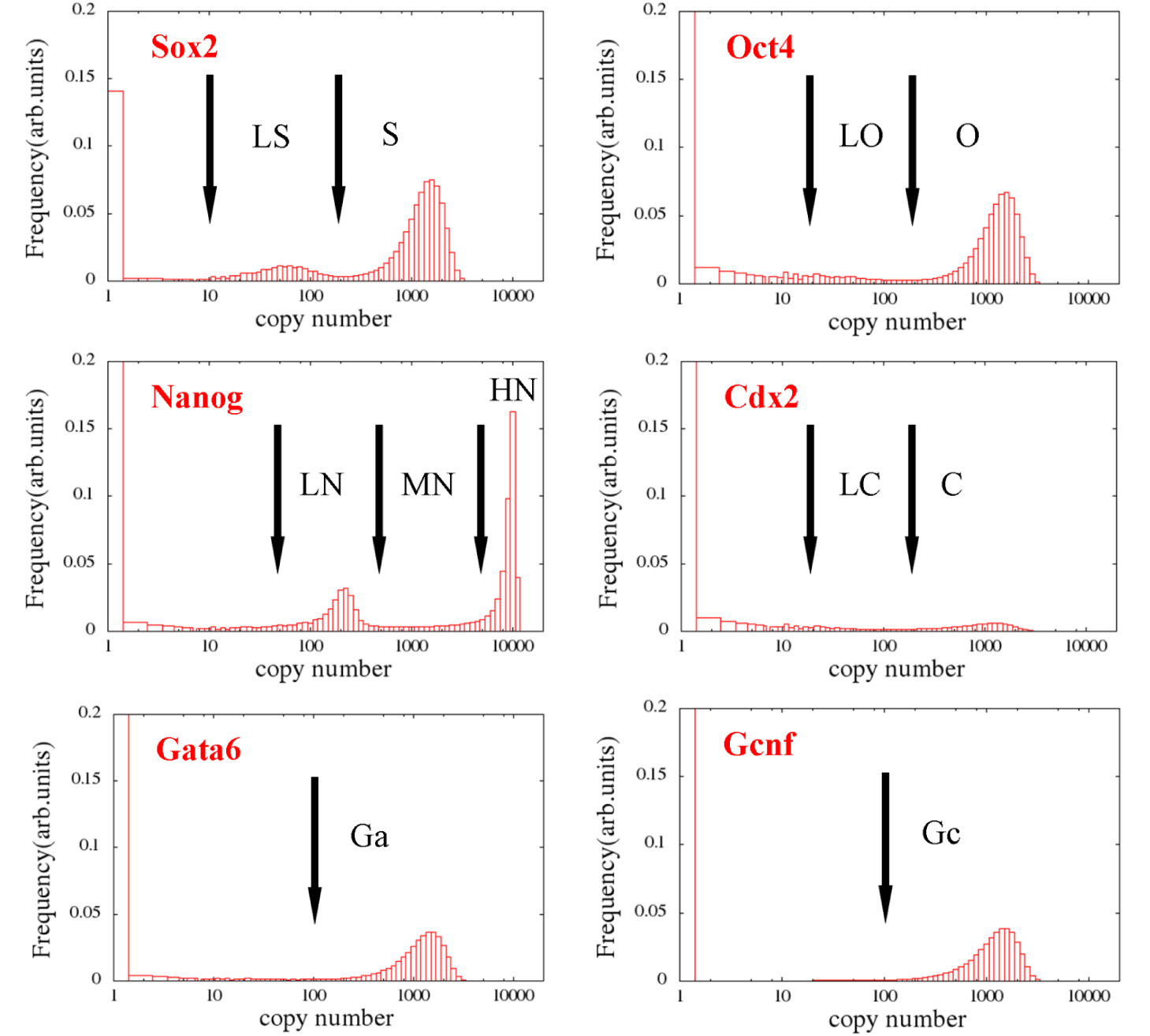

Supplement: Figure S2 — Simulated distributions of the copy number of protein factors which appeared in trajectories of the differentiation process from the ESC states to the Gata6-dominant state. Distributions are divided to define the cell states by introducing thresholds designated by arrows. The abbreviations used to refer the cell states in Fig. 6C of the main text are written on each panel. 10,000 trajectories for 11.5 days were used for sampling the data. (TIF) [file pcbi.1003380.s002.tif]

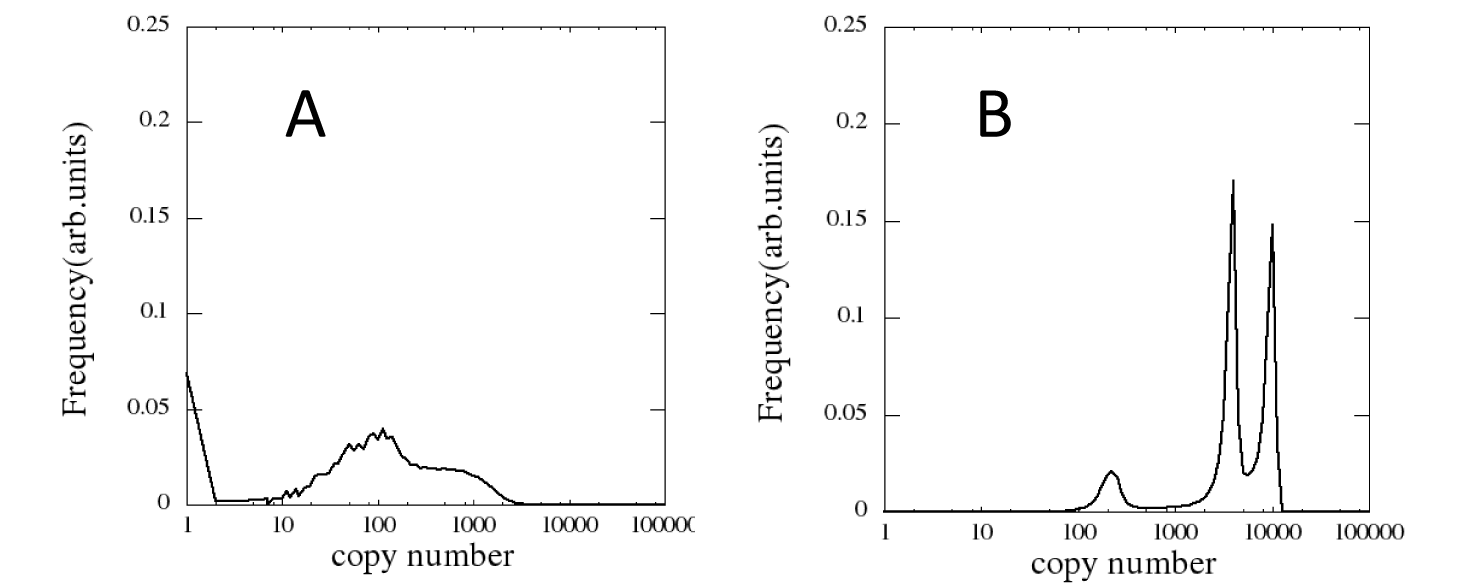

Supplement: Figure S3 — Distribution of expression level of Nanog in the case that either of two alleles of Nanog is not silenced through the allelic regulation. (A) and (B) . Other parameters are the same as those used in Fig. 3 of the main text. (TIF) [file pcbi.1003380.s003.tif]

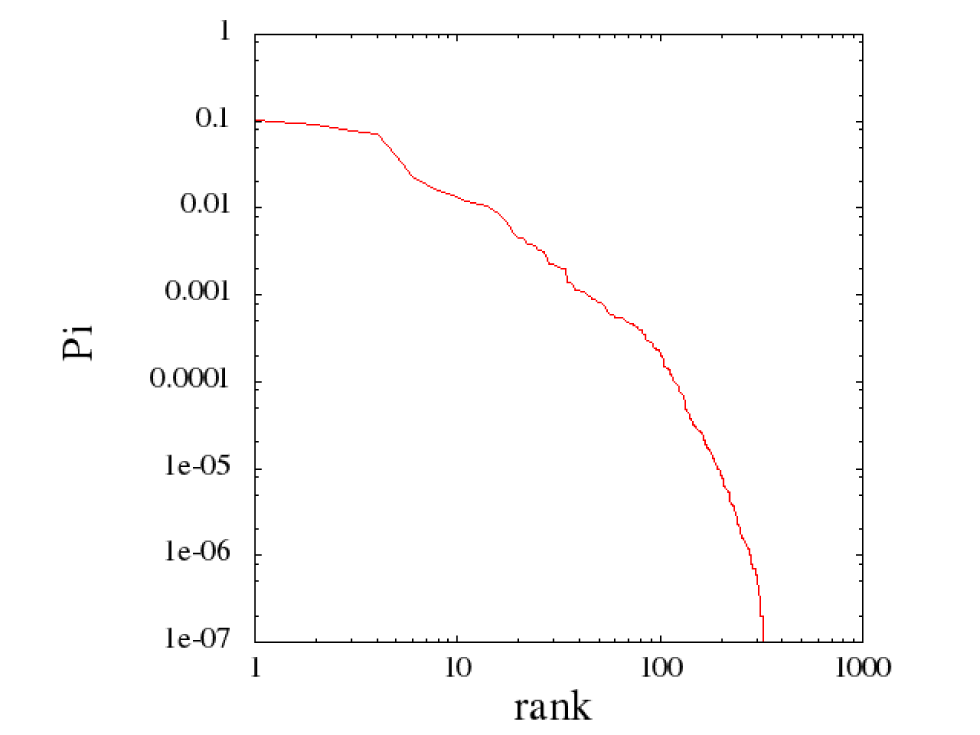

Supplement: Figure S4 — Probability of the appearance of the cell state in the 10,000 simulated trajectories are shown in the rank order of . (TIF) [file pcbi.1003380.s004.tif]
